# Genome-wide comparison of the protein-coding repertoire reveals fast evolution of immune-related genes in cephalochordates and Osteichthyes superclass

**DOI:** 10.18632/oncotarget.22749

**Published:** 2017-11-28

**Authors:** Qi-Lin Zhang, Bin Xu, Xiu-Qiang Wang, Ming-Long Yuan, Jun-Yuan Chen

**Affiliations:** ^1^ LPS of Nanjing Institute of Geology and Palaeontology, CAS, Nanjing, China; ^2^ State Key Laboratory of Pharmaceutical Biotechnology, School of Life Science, Nanjing University, Nanjing, China; ^3^ State Key Laboratory of Grassland Agro-Ecosystems,College of Pastoral Agriculture Science and Technology, Lanzhou University, Lanzhou, China

**Keywords:** cephalochordates, Osteichthyes superclass, immune-related genes, fast evolution, protein-coding genes, Immunology

## Abstract

Amphioxus is used to investigate the origin and evolution of vertebrates. To better understand the characteristics of genome evolution from cephalochordates to Osteichthyes, we conducted a genome-wide pairwise comparison of protein-coding genes within amphioxus (a comparable group) and parallel analyses within Osteichthyes (two comparable groups). A batch of fast-evolving genes in each comparable group was identified. Of these genes, the most fast-evolving genes (top 20) were scrutinized, most of which were involved in immune system. An analysis of the fast-evolving genes showed that they were enriched into gene ontology (GO) terms and pathways primarily involved in immune-related functions. Similarly, this phenomenon was detected within Osteichthyes, and more well-known and abundant GO terms and pathways involving innate immunity were found in Osteichthyes than in cephalochordates. Next, we measured the expression responses of four genes belonging to metabolism or energy production-related pathways to lipopolysaccharide challenge in the muscle, intestine or skin of *B. belcheri*; three of these genes (*HMGCL*, *CYBS* and *MDH2*) showed innate immune responses. Additionally, some genes involved in adaptive immunity showed fast evolution in Osteichthyes, such as those involving “intestinal immune network for IgA production” or “T-cell receptor signaling pathway”. In this study, the fast evolution of immune-related genes in amphioxus and Osteichthyes was determined, providing insights into the evolution of immune-related genes in chordates.

## INTRODUCTION

**Figure 1:**
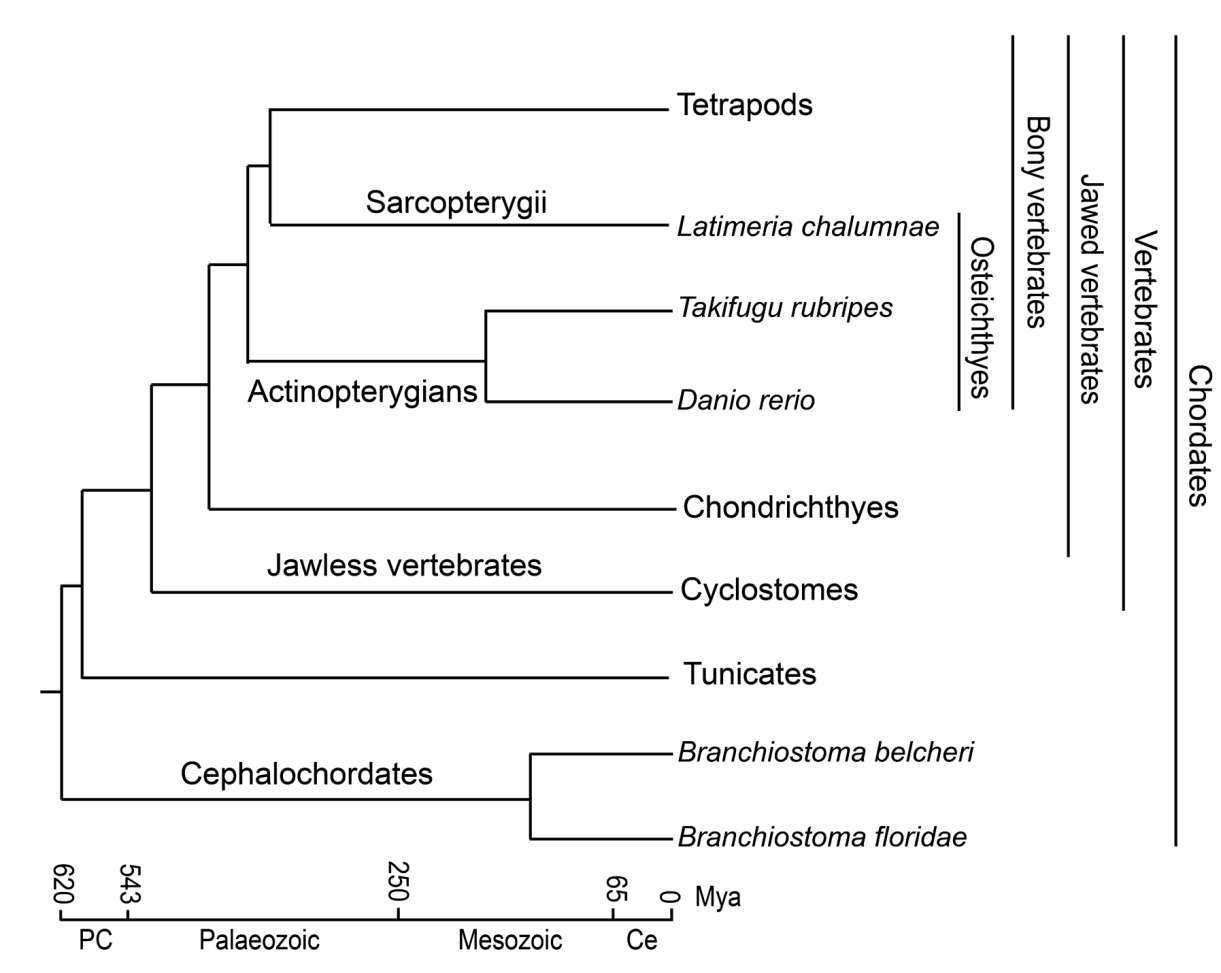
Phylogenetic tree and evolutionary time frame for chordates Adapted from Yue et al. and Venkatesh et al. [2, 7].

Generally, jawed vertebrates, such as Osteichthyes and mammals, have developed an elaborate adaptive immune system with diversified B-cell and T-cell antigen receptors (BCRs and TCRs) [1, 2]. Yet, jawless vertebrates, such as cyclostomes, have variable lymphocyte receptors (VLRs) with somatically rearranged LRR ectodomains [3, 4]. Therefore, the adaptive immunity may be traced to an early stage of vertebrate evolution (Figure 1). A recent study found that primordial recombination-activating gene (protoRAG) in amphioxus could correctly splice mRNA and conduct actual transposition in human cells [5]. Cao et al. reported the structure of a VLR-like gene (similar to VLR-c type) and preliminarily detected innate immune function of this gene in amphioxus [6]. However, antigen receptors with naturally adaptive immune activity have not been found in amphioxus. Therefore, the assumption is that cephalochordates strongly rely on the innate immune system according to our current knowledge [1, 7].

Amphioxus, the only modern representative of the subphylum Cephalochordata, has long been considered the extant invertebrate most closely related to the proximate invertebrate ancestor of vertebrates [7-9]. Amphioxus is a model organism widely used in comparative genomics, developmental homology of vertebrates and comparative immunology studies [10]. Generally, model amphioxus primarily includes three genera (*Branchiostoma, Epigonichthys* and *Asymmetron*), with most species (over 28 of ∼31 species) in *Branchiostoma* [7], which has a wide distribution from low to high latitudes [11]. For the tropical amphioxus *Asymmetron*, two recognized species are primarily distributed along low latitudes [12], whereas only one *Epigonichthys* species is recognized [7]. Yue et al. reported that many fast-evolving genes between *Branchiostoma* and *Asymmetron* were associated with innate immunity [7]. However, two *Branchiostoma* species (*B. floridae* and *B. belcheri*) separated from the common ancestor ∼120 million years ago (Mya) (Figure 1) [7]. Moreover, *Branchiostoma* has a wide distribution, indicating that *Branchiostoma* species may have experienced more diverse and complex environments than the other two genera in amphioxus. Thus, fast-evolving genes have evolved functional divergence between *Branchiostoma* and *Asymmetron*, which would be different from that of the interspecific divergence of fast-evolving genes within *Branchiostoma*. Within the completed *Branchiostoma* genome (*floridae* and *belcheri*) sequence [1, 9], amphioxus exhibits complexity and diversity of the gene repertoire for innate immunity [1]. However, evolutionary characteristics of innate immunity in the speciation of amphioxus remain largely unknown, impeding our understanding of the evolution of innate immune-related genes in amphioxus.

Previous studies showed that the emergence of mmunoglobulin-based adaptive immunity accompanied the origin of jawed vertebrates (gnathostomes) [2] (Figure 1). However, the adaptive immune system of cartilaginous fish (Chondrichthyes, the original jawed vertebrates) lacks key transcription factors, such as the cluster of differentiation 4 (CD4), RAR related orphan receptor C (RORC), and most CD4-lineage-specific cytokines and cytokine receptors, suggesting that adaptive immunity is more complete in bony vertebrates with the acquisition of helper and regulatory functions of T-cells that recognize MHC class II molecules [2]. Osteichthyes superclass, the oldest group in bony vertebrates (Figure 1), have developed more elaborate adaptive immune systems [2]. This animal group is composed of lobe-finned fish (Sarcopterygii) and ray-finned fish (Actinopterygii). The African coelacanth (*Latimeria chalumnae*) is a rare existing in Coelacanthimorpha in Sarcopterygians [13] (Figure 1). Because of a wide distribution and high adaptability and diversity, ray-finned fish are important experimental materials in studies of genetics and immunity [14-16]. The zebrafish (*Danio rerio*) and pufferfish (*Takifugu rubripes*) are two kinds of fish that are well-known model animals with sequenced genomes that are the subjects of many comparative studies conducted to explore genome evolution, gene function, and development and to discover immune- and disease-related genetic resources [17-20].

Here, we ask scientific questions: Within *Branchiostoma*, are innate immune-related genes fast-evolving? Next, whether this situation exists within Osteichthyes? Finally, what difference of immune-related genes in Osteichthyes can be obtained based on functional enrichments of fast-evolving gene sets? First, we performed comparative analyses between *B. floridae* and *B. belcheri* (Bf *vs* Bb) to identify fast-evolving gene sets (including particularly fast-evolving genes, PFEGs, and moderately fast-evolving genes, MFEGs); second, enrichment of functional terms (Gene ontology, GO) of PFEGs and MFEGs was determined to explore their potential functions; finally, to detect pathways that contained fast-evolving genes, we determined significantly enriched pathways (as defined in Kyoto Encyclopedia of Genes and Genomes, KEGG) for MFEGs. Furthermore, two parallel analyses for fast-evolving gene sets were conducted for *Danio rerio vs*. *Latimeria chalumnae* (Dr *vs* Lc) and *D. rerio vs*. *Takifugu rubripes* (Dr *vs* Tr) (these animals are in basal or advanced evolutionary nodes of Crossopterygii or Actinopterygii in Osteichthyes), respectively. To observe whether PFEGs (enriched KEGG pathways associated with metabolism and energy production) possessed innate immune activity in amphioxus, we used quantitative real-time PCR (qRT-PCR) to detect the expression responses of four genes in skin, intestine or muscle of *B. belcheri* challenged with lipopolysaccharide (LPS). The results of this study will provide some broader insights into the evolution of immune-related genes in cephalochordates and Osteichthyes.

## RESULTS

### Sequence annotation, putative orthologous genes and test for selection pressure

Statistics for the annotation information of the gene sets used in this study is shown in Supplementary Table 1. We obtained 15,008, 12,645 and 14,475 orthologous gene pairs in Bf *vs* Bb, Dr *vs* Lc and Dr *vs* Tr, respectively. After trimming and gap filtration, the alignment coverage of each of the orthologous gene pairs in these three comparable groups was ≥ 80%, and the range of the alignment lengths was from 138 to 22,551 bp in Bf *vs* Bb, 129 to 21,705 bp in Dr *vs* Lc and 141 to 19,671 bp in Dr *vs* Tr. Table 1 shows the values of *Ka*, *Ks* and *Ka/Ks* across all genes for the three comparable groups. For the Bf *vs* Bb, we removed 4,393 invalid genes and the remaining genes were used in further analyses based on *Ka/Ks* values. Regarding the Dr *vs* Lc and Dr *vs* Tr sets of orthologous genes that we used, more than 50% of orthologous gene pairs had values of *Ks* > 1 (genome-wide average *Ks* > 3, Table 1). Thus, we only considered the *Ka* values as the criteria to scale evolutionary rates in Dr *vs* Lc and Dr *vs* Tr. Independent of *Ka/Ks* or *Ka* values that we used when identifying the PFEGs, the PFEG sets showed a 2.5- to 3.5-fold higher evolutionary rates than that of the genome-wide average (Table 1). We found a 2.1- to 3.2-fold higher evolutionary rate in MFEG sets than that of the genome-wide average under more relaxed criterion.

**Table 1:** Means of genome-wide Ka, Ks, and Ka/Ks values and statistics of GO and KEGG terms for three comparable groups.

	All genes			Particularly fast-evolving genes (top 5% identified by Ka)			Fast-evolving genes (top 10% identified by Ka)			Particularly fast-evolving genes (top 5% identified by Ka/Ks)	Fast-evolving genes (top 15% identified by Ka/Ks)
	Bf vs Bb	Dr vs Lc	Dr vs Tr	Bf vs Bb	Dr vs Lc	Dr vs Tr	Bf vs Bb	Dr vs Lc	Dr vs Tr	Bf vs Bb	Bf vs Bb
*Ka*	0.3033	0.2826	0.2042	1.0338	0.6965	0.5756	1.0174	0.6341	0.5003	1.0307	1.0174
*Ks*	0.8714	3.2931	3.3977	0.8963			0.9459			0.8936	0.9459
*Ka/Ks*	0.3162			1.1584			1.0804			1.1586	1.0804
T^#^				130	21	32	227	23	61	138	166
N*				87	22	25	147	20	40	92	112
N/T (%)				66.92	95.65	78.13	64.76	95.24	65.57	66.67	67.47
K^&^							20	31	37		19

^#^ T: Total number of GO terms for which the particular gene sets are significantly enriched; *N: Number of immune-related GO for which the particular gene sets are significantly enriched; ^&^K: Number of KEGG terms for which the particular gene sets are significantly enriched. *Branchiostoma floridae* vs *B. belcheri* (Bf vs Bb); *Danio rerio* vs. *Latimeria chalumnae* (Dr vs Lc); *D. rerio* vs. Takifugu rubripes (Dr vs Tr).

### Analysis of fast-evolving genes

The top 20 fast-evolving genes in three comparable groups are presented in Table 2. In Bf *vs* Bb, the most fast-evolving genes encoded an iron-sulfur cluster assembly scaffold protein (ISCU). Additionally, seven fast-evolving genes encoded typical immune-related proteins, including Toll-like receptor 4 (TLR4), interleukin-17 receptor D (IL17RD), complement component C1q receptor (CD93), complement factor H-related protein 1 (CFHR1), interferon regulatory factor 4 (IRF4), TNF receptor-associated factor 6 (TRAF6), NACHT, LRR and PYD domains-containing protein 3 (NLRP3). The most fast-evolving genes in Dr *vs* Lc encoded mucin-1 (MUCIN1). A large number of fast-evolving genes encoded typical immune-related proteins were detected, such as macrophage receptor with collagenous structure (MARCO), interleukin 2 receptor, beta (IL2RB), hematopoietic death receptor (HDR), and so on. In Dr *vs* Tr, the most fast-evolving genes encoded an interleukin 13 receptor, alpha 1 (IL13RA1), followed by lymphotoxin alpha (LTA). Moreover, three fast-evolving genes encoded cytokine receptor family member b2, b1, b6 (CRFB2, 1, 6); six fast-evolving genes encoded interleukin-related proteins, including interleukin 20 receptor, alpha (IL20RA), interleukin 21 (IL21), interleukin 15, like (IL15L), interleukin-1 family member A (IL1FMA), interleukin 2 receptor, beta (IL2RB), interleukin 12a (IL12A). Notably, CD79b molecule, immunoglobulin-associated beta (CD79B) involving B-cell antigen receptor function, was identified.

**Table 2:** Top 20 most fast-evolving genes in each of three comparable groups.

Gene description (*B. floridae* versus *B. belcheri* )	*Ka/Ks*	Gene name
Iron-sulfur cluster assembly scaffold protein IscU	1.5847	ISCU
Ultraviolet-B receptor UVR8	1.5294	UVR8
Toll-like receptor 4	1.4546	TLR4
Interleukin-17 receptor D	1.4422	IL17D
DNA-directed RNA polymerase II subunit RPB7	1.4417	POLR2G
Vacuolar ATPase assembly integral membrane protein VMA21	1.4351	VMA21
Complement component C1q receptor	1.4237	CD93
E3 ubiquitin-protein ligase TRIM71	1.4069	TRIM71
Transforming growth factor-beta-induced protein	1.3847	TGFBI
Complement factor H-related protein 1	1.3837	CFHR1
UBA-like domain-containing protein 2	1.3790	UBALD2
Interferon regulatory factor 4	1.3760	IRF4
Thymosin beta-10	1.3751	TMSB10
Polycomb group RING finger protein 1	1.3662	PCGF1
Astrocytic phosphoprotein PEA-15	1.3645	PEA15
Cytochrome c2	1.3574	C2
TNF receptor-associated factor 6	1.3521	TRAF6
Transmembrane protein 203	1.3421	TMEM203
Sperm-associated antigen 7	1.3412	SPAG7
NACHT, LRR and PYD domains-containing protein 3	1.3376	NLRP3
**Gene description (*D. rerio* versus *L. chalumnae*)**	***Ka***	**Gene name**
Mucin-1	0.9894	MUCIN1
Chromogranin B	0.9799	CHGB
Macrophage receptor with collagenous structure	0.9249	MARCO
Podocalyxin-like 2	0.8992	PODXL2
Meis homeobox 1 b	0.8945	MEIS1
Eph receptor A3	0.8915	EPHA3
MACRO domain containing 1	0.8908	MACROD1
Notch 2	0.8753	NOTCH 2
Complement factor H like 4	0.8690	CFHL4
Spalt-like transcription factor 1a	0.8690	SALL1A
Myelin transcription factor 1a	0.8648	MYT1A
CD79a molecule, immunoglobulin-associated alpha	0.8630	CD79a
Mitogen-activated protein kinase kinase kinase 5	0.8609	MAP3K5
Interleukin 2 receptor, beta	0.8536	IL2RB
Hematopoietic death receptor	0.8523	HDR
NLR family, CARD domain containing 3	0.8498	NLRC3
Calcitonin/calcitonin-related polypeptide, alpha	0.8456	CALCA
Caspase 8 associated protein 2	0.8445	CASP8AP2
MAP7 domain containing 2a	0.8443	MAP7D2A
Nuclear factor of kappa light polypeptide gene enhancer in B-cells inhibitor, zeta	0.8405	NFKBIZ
**Gene description (*D. rerio* versus *T. rubripes*)**	***Ka***	**Gene name**
Interleukin 13 receptor, alpha 1	1.1271	IL13RA1
lymphotoxin alpha (TNF superfamily, member 1)	0.9460	LTA
Telomeric repeat binding factor a	0.8847	TERFA
Secreted phosphoprotein 1	0.8533	SPP1
Cytokine receptor family member b2	0.8403	CRFB2
Neurotrophic tyrosine kinase, receptor, type 2a	0.8241	NTRK2A
Serine/threonine/tyrosine interacting-like 1	0.8119	STYXL1
Interferon-induced protein with tetratricopeptide repeats 15	0.8108	IFIT15
Interleukin 20 receptor, alpha	0.8070	IL20RA
CD79b molecule, immunoglobulin-associated beta	0.8065	CD79B
Polymerase (DNA directed) kappa	0.8056	POLK
Interleukin 21	0.8042	IL21
Cytokine receptor family member b1	0.7987	CRFB1
Interleukin 15, like	0.7981	IL15L
Interleukin-1 family member A	0.7902	IL1FMA
Interleukin 2 receptor, beta	0.7902	IL2RB
Interleukin 12a	0.7884	IL12A
Collagen, type IV, alpha 3	0.7844	COI4A3
Threonine synthase-like 2	0.7801	THNSL2
Cytokine receptor family member b6	0.7744	CRFB6

### Analysis of functional classes of fast-evolving genes

We found that 130 and 138 GO categories were significantly enriched for PFEG sets in Bf *vs* Bb based on the values of *Ka* and *Ka/Ks*, respectively (Table 1, Supplementary Tables 2 and 3); for the MFEG sets, 227 (top 10% *Ka*) and 166 (top 15% *Ka/Ks*) terms were significantly enriched. Most GO terms (> 80%) enriched in the PFEG set were included in those enriched in the MFEG sets (Supplementary Tables 2 and 4). Additionally, for MFEG sets in Bf *vs* Bb, more than 90% of GO terms identified by *Ka/Ks* overlapped with terms identified by *Ka* (Supplementary Tables 4 and 5). We found that 21 and 23 GO terms were overrepresented in the PFEG and MFEG sets of Dr *vs* Lc, respectively (Table 1, Supplementary Tables 6 and 7). For Dr *vs* Tr, 32 and 61 GO categories were significantly enriched in the PFEG and MFEG sets, respectively (Table 1, Supplementary Tables 8 and 9). GO terms enriched in the MFEG set included most of GO terms (> 70%) enriched in the PFEG set in both Dr *vs* Lc and Dr *vs* Tr.

Among enriched GO terms for Bf *vs* Bb, most were associated with the neural/immune category. For example, 66.92% of GO terms identified by the top 5% *Ka* and 66.67% by the top 5% *Ka/Ks* values were included in the list of neural/immune-related GO terms [21]. Additionally, 64.76% and 67.47% of GO terms identified by the top 10% *Ka* and the top 15% *Ka/Ks* values, respectively, were detected in this curated collection [21]. The nervous and immune systems are well known to interact with each other; the function of the immune system is modulated by the nervous system, which in turn influences nervous system [22-24]. In the analysis of Dr *vs* Lc, the enrichment of neural/immune-related functional GO terms (both innate and adaptive) by fast-evolving genes was more obvious than that of Bf *vs* Bb and Dr *vs* Tr (Table 1). A relatively fast evolution of immune-related genes was also observed in other fish, including the zebrafish and grass carp (*Ctenopharyngodon idellus*) [25], large yellow croaker (*Larimichthys crocea*) and Atlantic cod (*Gadus morhua*) [14, 26]. Many of the enriched GO terms for fast-evolving genes identified by Bf *vs* Bb were related to transmembrane cellular function, signal activity and receptor activity associated with response to stimuli, indicating potential roles in innate immunity. The most compelling GO categories were those involved with the mitochondrial membrane, such as “mitochondrial part”, “mitochondrial inner membrane”, “mitochondrial membrane”, “mitochondrial envelope”, “mitochondrial respiratory chain” and “mitochondrial membrane part” (Supplementary Tables 2-5). Many immune-related genes are located in mitochondrial membranes and mitochondria and are part of the respiratory chains that are key in the innate immune system [27, 28]. Additionally, several well-known immune genes of amphioxus were also detected in fast-evolving gene sets, such as the NOD-like receptor (NLR) genes, fork-head transcription factor (FOX) gene and IkB kinase gene. Furthermore, the enriched GO categories for fast-evolving genes in Dr *vs* Lc and Dr *vs* Tr were primarily related to immune/defense response, cytokines, receptor activity and cell death. In particular, the GO terms involved in adaptive immunity were detected in the comparable groups within bony fish and included terms (i.e. “MHC protein complex”) which functioned in the recognition of cell surfaces for T-cells (Supplementary Tables 7 and 8).

### Analysis of pathways of fast-evolving genes

**Figure 2:**
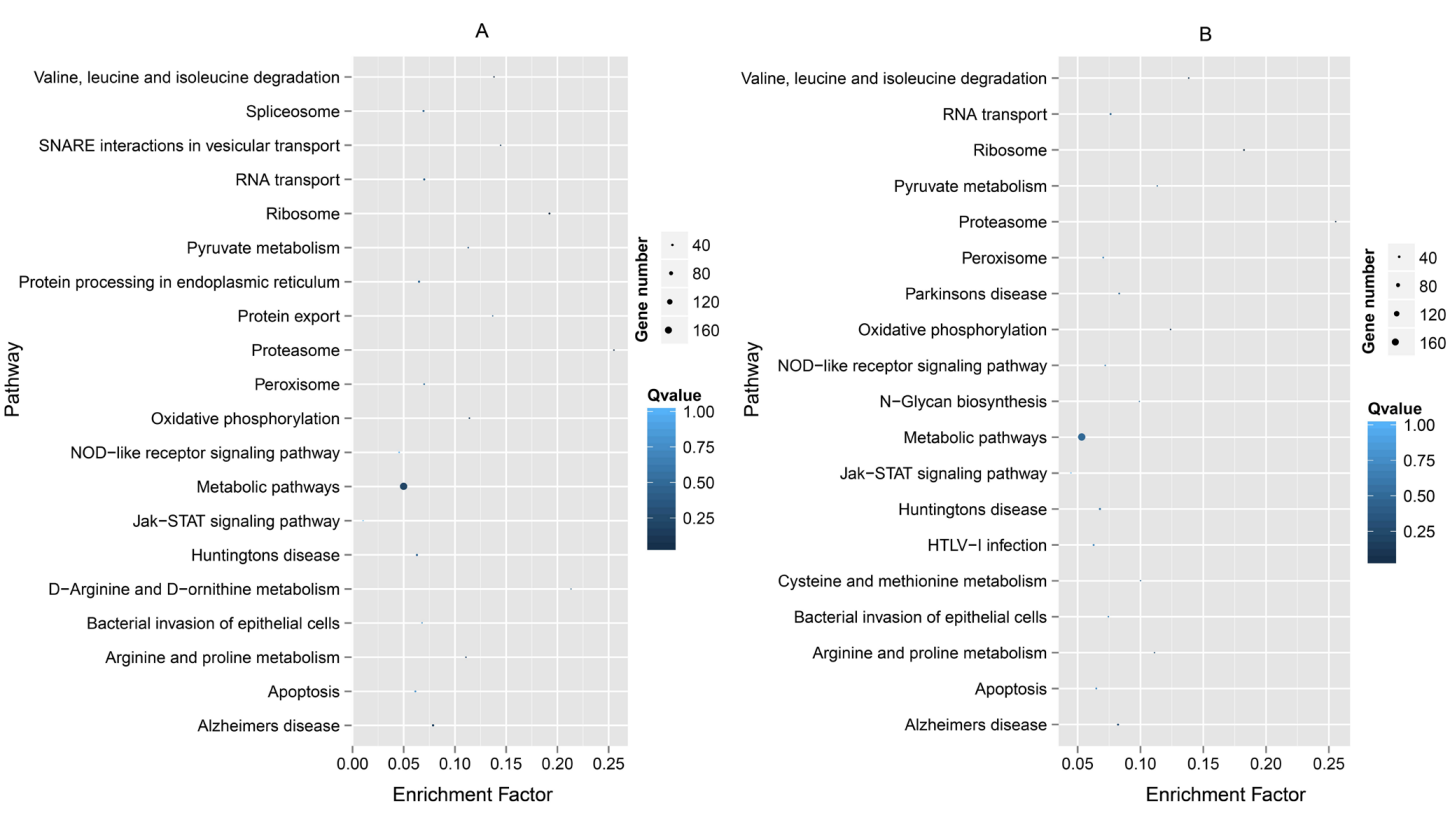
Top 20 KEGG pathways enriched by the top 10% Ka **(A)** and the top 15% Ka/Ks **(B)** for Branchiostoma belcheri *vs* B. floridae in descending order of the *P*-values. A low *P*-value indicates a high level of enrichment.

**Figure 3:**
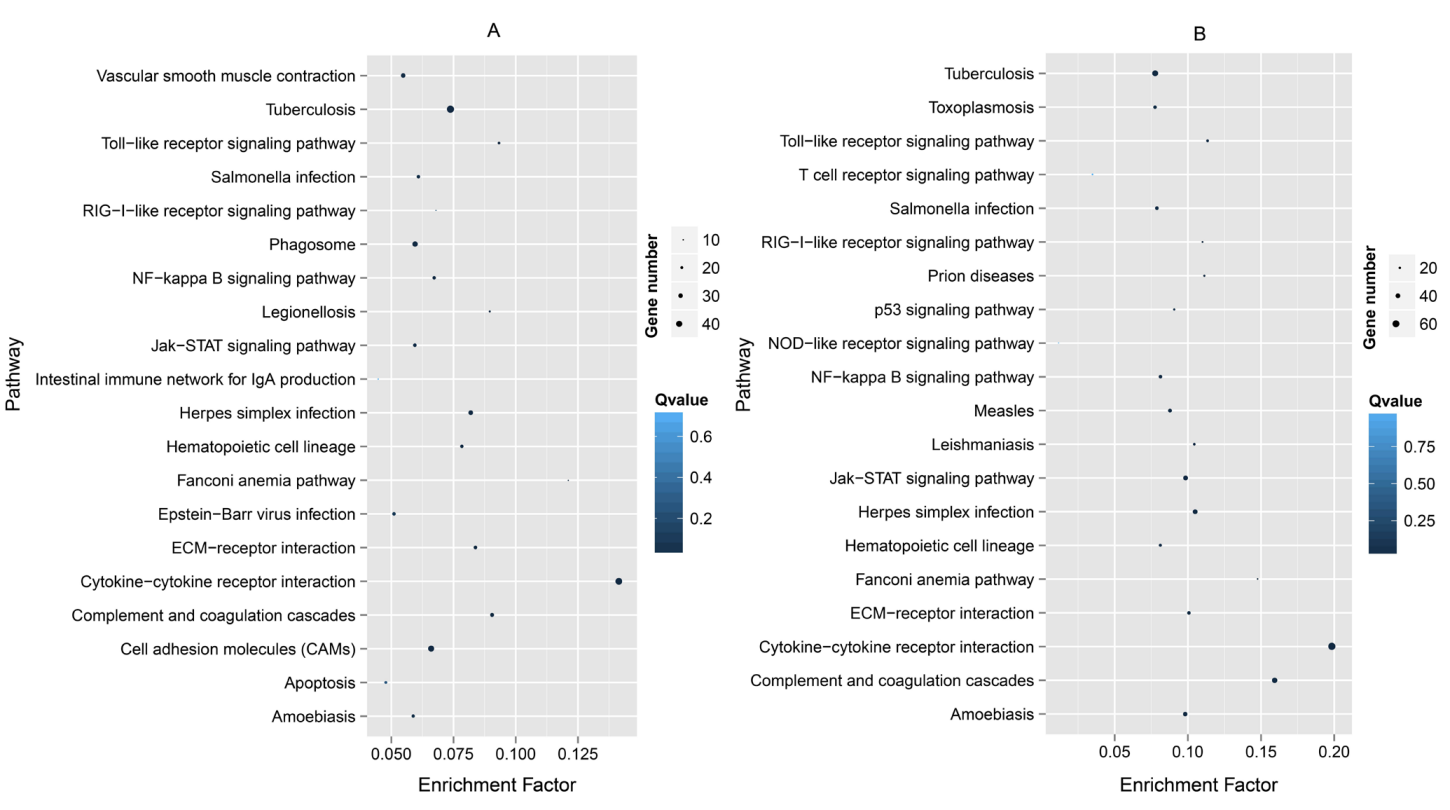
Top 20 KEGG pathways enriched by the top 10% Ka for Latimeria chalumnae *vs* Danio rerio **(A)** and for Takifugu rubripes *vs* D. rerio **(B)** in descending order of the *P*-values.

Fast-evolving genes in Bf *vs* Bb, the top 10% *Ka* and 15% *Ka/Ks* values were classified significantly into 20 and 19 KEGG pathways, respectively (Table 1). The results showed some immune- and disease-related pathways, such as the proteasome, Jak-STAT signaling pathway, the peroxisome, bacterial invasion of epithelial cells and NOD-like receptor signaling pathway (Figure 2). Some well-known pathways involved in energy production and amino acid metabolism were notable. For Dr *vs* Lc and Dr *vs* Tr, the significant results of enriched KEGG pathways for the MFEG set had 31 and 37 terms (Table 1), respectively. When the top 20 KEGG categories were plotted based on descending order of the significance level, we found that almost all pathways were related to a response to immune stimulation (Figure 3). These pathways primarily involved cell surface receptors in innate immunity, infections and immune diseases, cytokines, apoptosis and adaptor molecules in immune signaling pathways. Particularly, we detected “T-cell receptor signaling pathway”, “intestinal immune network for IgA production” and “hematopoietic cell lineage”, indicating that some of the genes associated with adaptive immunity were fast evolving in Dr *vs* Tr.

### Expression of *HMGCL*, *SAT2*, *CYBS* and *MDH2* challenged with lipopolysaccharide

**Figure 4:**
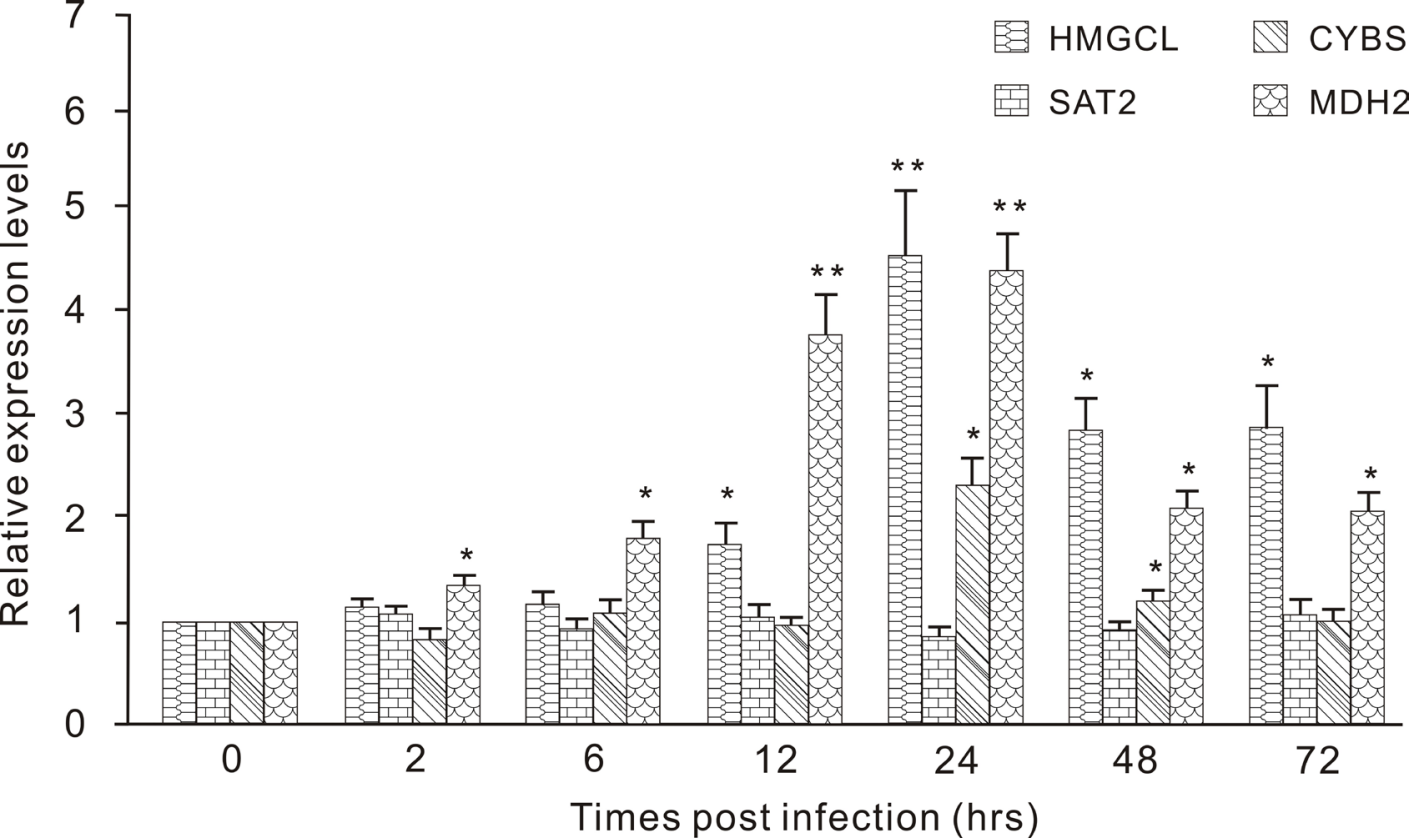
Expression profiles of HMGCL, SAT2, CYBS and MDH2 in the different tissues (skin, intestine and muscle) of Branchiostoma belcheri challenged with LPS *HMGCL* in the skin, *SAT2* in the intestine, and *CYBS* and *MDH2* in the muscle Expression level at each time point is shown as the mean ± SD (*n* = 3). “**” highly significant difference compared with the control, *P* < 0.01; “*”significant difference, *P* < 0.05.

To determine the innate immune activity of the fast-evolving genes in metabolism or energy production-related pathways, we measured the expression of four of these genes in the muscle, intestine or skin of *B. belcheri* challenged by a LPS. The expression responses of four types of genes to LPS stimulation were determined over a 72 hour time course: mitochondrial hydroxymethylglutaryl-CoA lyase (*HMGCL*) in skin, diamine acetyltransferase 2 (*SAT2*) in the intestine, and succinate dehydrogenase cytochrome B small subunit precursor (*CYBS*) and mitochondrial malate dehydrogenase (*MDH2*) in muscle (Figure 4). Upon a LPS simulation, only one expression peak was detected for *HMGCL* at 6 hours post infection (hpi) with a 4.4-fold increase compared with the control, and the level of expression was significantly higher than that from 12 to 72 hpi. For *SAT2*, no significant increase was detected at any hpi. In this experiment, we found that *CYBS* only showed significantly higher expressions at 24 and 48 hpi than that in the control. Regarding the expression profile for *MDH2*, we detected an initial, significant up-regulation at 2 hpi, then reached a single expression peak in muscle at 24 hpi when the expression was 4.3-fold higher than that of the control. At the two later time points, the expression of *MDH2* continued to be up-regulated, compared with that of the control.

## DISCUSSION

In the absence of an adaptive immune system, amphioxus primarily depends on a diverse and complex innate immune system to provide protection against viral and microbial pathogens in seawater [1, 7]. Huang et al. found that many classical innate immune-related gene repertoires experienced significant expansion in *B. floridae*, including cell surface receptors (Toll-like receptors, TLRs; NLRs), scavenger receptors (SRCRs), caspases, adaptors and components of the complement system [1]. Some domains have undergone strongly positive selection in expansion, such as leucine-rich repeats (LRRs) [1]. Under gene expansion and domain shuffling, the innate immune system that developed in amphioxus was even shaped by altering the topology of protein-protein networks [1, 29]. Therefore, the high proportion of fast-evolving genes involved in innate immunity observed in our results was not unexpected. In particular, based on this exploration, improvement in the immune system likely relied on rapid variation in the sequences and copies of innate immune-related genes; perhaps, this fast evolution of sequences was an important factor in the expansion of genes, as reported in ticks (*Ixodes scapularis*) [30, 31]. Among the top 20 fast-evolving genes, we found many typical immune-related genes in innate immune system, i.e. cytokines, pattern recognition receptor (PRRs), complement and signal transducers. Some well-known pathways with annotated information were involved in metabolism and energy production in enriched KEGG terms of Bf *vs* Bb, such as “metabolic pathway” and “oxidative phosphorylation”. This result was not completely unexpected because several neural/immune GO terms were also annotated to this information, such as “metabolic process” and “oxidation-reduction process” (Figure 2). Additionally, many studies showed that several metabolism-related genes and pathways played key roles in innate immunity [32-34]. In particular, genes associated with metabolism and energy production usually experienced fast evolution among groups (ecotypes) living in habitats with extreme environmental differences (e.g., fish living at high and low elevations, facing severe cold and heat, and inhabiting marine and fresh water) [35, 36]. Nevertheless, amphioxus are generally found half-buried in sand (with much less movement than jawed vertebrates) between 5 and 10 meters below sea level, with a distribution in moderate temperature ranges (from shallow temperate to tropical seas) [37]. Thus, the enriched pathways with annotation information involved in metabolism and energy most likely participated in innate immunity. Additionally, three PFEGs (*HMGCL*, *CYBS* and *MDH2*) enriched in metabolic and energy production-related pathways showed an expression response to LPS, further supporting our contention. However, most innate immune activity of these genes commonly appeared at 24 hpi, suggesting possible indirect roles in innate immunity. *SAT2* did not show a response in the intestine challenged with LPS, indicating that some members associated with these pathways might indeed function primarily to meet metabolism and energy requirements. However, this relation requires further confirmation in more challenged tissues with more post-infection time points, number of genes and types of stimuli. Overall, many fast-evolving genes were related to innate immunity within *Branchiostoma*. Our study provided evolutionary characteristics of the interspecific divergence of immune-related genes within the same amphioxus genus. Moreover, previous studies have reported that many fast evolving genes were involved in innate immunity between *Branchiostoma* and *Asymmetron* [7], therefore we proposed that this phenomenon may be common in cephalochordates.

In addition, in our parallel analyses of Dr *vs* Lc and Dr *vs* Tr, several GO terms that were primarily enriched involved cytokines, immune processes, defense, receptor signaling pathways and apoptosis. Both within Actinopterygii (Dr *vs* Tr) and between Actinopterygii and Crossopterygii (Dr *vs* Lc), most of enriched GO terms were related to immunity. Additionally, we also detected many typical innate immune-related genes among the top 20 fast-evolving genes in these two comparable groups, of which genes involving interleukin function were found much more frequently and the other genes associated with complement, PRRs, cytokines, signal transducers and caspases were also well-know typical innate immune components. However, Osteichthyes have developed a more elaborate adaptive immune system, which includes major histocompatibility complex (MHC), immunoglobulins (IGs), costimulatory molecules and relevant cytokines [2, 38]. In this study, we detected some genes involving adaptive immunity, including CD79b molecule, immunoglobulin-associated beta (CD79B), and lymphotoxin alpha (LTA), in the list of the top 20 fast-evolving genes. In addition, many cytokines and adaptors play key roles in both innate and adaptive immunity [38, 39]. Therefore, the enriched GO terms only indicated that many fast-evolving genes in Osteichthyes were involved in immunity, and further analyses will be required to confirm whether these genes are related to innate or adaptive immunity. Notably, most KEGG terms enriched by fast-evolving genes in Dr *vs* Lc were highly similar to those in Dr *vs* Tr, and some of these are well known to participate in innate immunity, including “Toll-like receptor signaling pathway”, “Complement and coagulation cascades”, “NOD-like receptor signaling pathway”, and “RIG-I-like receptor signaling pathway”. Thus, genes involved in innate immunity had a high proportion of the fast-evolving genes, which showed a pattern of parallel evolution between Osteichthyes and cephalochordates. A highly open environment provided opportunities for aquatic pathogens and other microorganisms in natural aquatic systems to spread rapidly and widely [40, 41], and within variable microenvironments, populations of microorganisms could evolve rapidly [42]. Therefore, it was possible explanation that many of the genes involved in innate immunity were fast evolving.

Compared with cephalochordates, some lineage-specific genes, GO and KEGG terms involved in adaptive immunity were fast-evolving in Osteichthyes, such as genes encoding CD79B, the “MHC protein complex”, “T-cell receptor signaling pathway”, and “Intestinal immune network for IgA production” (Figure 3). Indeed, many components associated with adaptive immunity (i.e. immunoglobulin genes, adaptive immunity relevant cytokines and MHC genes) experienced a certain level of expansion in Osteichthyes [43, 44], which suggested that the functions of fast-evolving genes involved in immunity were more diverse in Osteichthyes than those in cephalochordates. Therefore, we hypothesized that improved ability of Osteichthyes to defend against pathogens may be due to a diverse and complex immune system and also an increase in the diversity of immune-related gene libraries with fast evolution (i.e., rapid evolution of adaptive immune-related genes played a key role). It is noteworthy that within Osteichthyes (between Crossopterygii and Actinopterygii), we found that the ratio of number of immune-related GO to total number of GO terms for which the particular gene sets are significantly enriched in Dr *vs* Lc was notably higher (∼95%) than that of Dr *vs* Tr (Table 1), indicating that immune-related genes tend to rapidly evolve in different intensity for different groups within Osteichthyes. In addition to those immune-related genes in cephalochordates, Osteichthyes also possessed many other fast-evolving genes involved in activity, proliferation of and immunity with B- and T- cells, immunoglobulin function, regulation of cytokines, leukocyte function, regulation of CD4 and CD8, and lymphocyte function. Moreover, in previously paralleled analysis in mammals, Yue et al. found that the GO terms enriched by fast-evolving genes in the placental–marsupial comparison (mouse *vs*. opossum) were highly and diversely involved in adaptive immunity (see Supplementary Tables 2 and 3 in [7]). Thus, compared with cephalochordates, Osteichthyes developed some fast-evolving genes associated with adaptive immunity to combat pathogens, but the diversity and complexity may be much less than that of mammals. Given that amphioxus and Osteichthyes are key models for investigating the evolution of chordate immune systems [8, 9, 38], the results of this study demonstrated fast evolution of immune-related genes in cephalochordates and Osteichthyes, and provided some useful information for understanding the evolutionary characteristics of immune-related genes in chordates.

## MATERIALS AND METHODS

### Downloading and annotation of protein-coding gene sets

The protein-coding sequences (CDSs) and corresponding protein sequences of each sampled species were downloaded from online databases (Supplementary Table 10). Considering alternative splicing in which multiple CDSs corresponded to a single gene, the longest CDS of each of the reference genes was extracted as a unique representative for the corresponding gene. The Basic Local Alignment Search Tool (BLAST) finds regions of local similarity between sequences. The software compares nucleotide or protein sequences to target databases to infer functional and evolutionary relationships between sequences (https://blast.ncbi.nlm.nih.gov/Blast.cgi). BLAST tool (ftp://ftp.ncbi.nlm.nih.gov/blast/) with a default E-value (10e-5) was used to annotate genes to the NCBI nonredundant (NR) protein, GO and KEGG databases.

### Construction of putative orthologs

We identified putative orthologs for each of the three comparable groups using Proteinortho (v5.15) [45], a reciprocal best hits based method that is suitable for large-scale analysis. If multiple genes from a species are detected, they are assigned into a co-orthologous group; next, we retained the first member as a unique representative of each co-orthologous group for further analyses. We extracted corresponding CDS sequences according to the output results of Proteinortho using custom Perl script. Then, nucleotide sequence of each orthologs CDS was aligned by PRANK with the “-codon” parameter [46], and aligned sequences with ambiguous alignments were removed. A manual check was also conducted to correct potential errors. Trimmed alignments that contained fewer than 20 codons were removed.

### Tests for selection pressure and selection of fast-evolving genes

For the ortholog sets obtained, we calculated the nonsynonymous substitution (*Ka*), synonymous substitution (*Ks*) and *Ka/Ks* values using the *KaKs_*calculator based on a maximum-likelihood (ML) method [47]. Then, invalid orthologous pairs (with *Ka* = 0, *Ks* = 0 or *Ks* > 1) were removed to avoid substitution saturation (mutations are in steady state) at the synonymous substitutions when analyses were further conducted based on *Ka/Ks* values [7]; genes with values of *Ks* > 1 were retained when the fast-evolving gene sets were defined by *Ka* [7]. Generally, a higher *Ka* or *Ka/Ks* value indicates a faster evolutionary rate [7, 48]. For Bf *vs* Bb, we defined genes with the top 5% *Ka* and *Ka/Ks* values as PFEGs according to the method of Yue et al. [7]. To ensure the robustness of this analysis and reliable results, we examined alternative *Ka* (top 10%) and *Ka/Ks* cutoffs (top 15%) (MFEGs) to determine whether enriched GO terms were sensitive to the different thresholds.

Additionally, when the number of genes with *Ks* > 1 (saturated values) was more than 40% in all orthologous pairs, which suggested that the evolutionary scale was too large between two species and that the saturated *Ks* values biased the estimated *Ka/Ks* ratio, we were required to mask out the *Ka/Ks* ratio [7]. *Ka* values are recommended as a superior proxy for the measurement of evolutionary rates in sorting genes, particularly under this condition; thus, we defined the fast-evolving genes based on *Ka* measurements according to previous methods [7, 48]. For Dr *vs* Lc and Dr *vs* Tr, two PFEG sets were identified using the top 5% *Ka* criteria. Alternatively, we also defined two MFEG (top 10% *Ka*) sets for the Dr *vs* Lc and Dr *vs* Tr. Our analyses reflected the divergence degree of orthologous pairs between two species and did not discriminate whether a given gene had a faster evolutionary rate in one species than in another.

### Enrichment for functional classes and pathways of fast-evolving genes

Enrichment for functional terms of the defined fast-evolving gene sets was implemented in the Blast2GO pipeline based on Fisher’s exact test [49]. Multiple test correction was conducted with the Benjamini and Hochberg (BH) methods using the *p.adjust* module in the R statistical software package to control the false discovery rate (FDR). The set of significantly enriched GO categories (FDR values *<*0.05) was further filtered using GO Trimming software (v2.0) to remove redundant categories [50]. Immune-related GO terms were identified according to the method of Yue et al. [7, 21]. Namely, GO terms enriched for in the defined PFEG and MFEG sets were included in a list of neural/immune-related GO terms [21], which were considered as the target GO categories. Additionally, enriched analyses for KEGG pathways of the PFEG and MFEG sets were performed using KOBAS 2.0 [51], and the pathways with *P*-values *<* 0.05 were considered as significantly enriched targets.

### Collection of experimental tissues of amphioxus

To explore whether genes in the metabolic and energy production-related KEGG pathways enriched in fast-evolving gene sets had innate immune activity, we selected the following four genes with different evolutionary rates in the sets that only contained genes with *Ka/Ks* > 1: *HMGCL* (higher rate, *Ka/Ks =* 1.38, in “Metabolic pathways”, ko01100), *SAT2* (high rate, *Ka/Ks =* 1.14, in “Arginine and proline metabolism”, ko00330), *CYBS* (moderate rate, *Ka/Ks =* 1.09, in “Oxidative phosphorylation”, ko00190) and MDH2 (low rate, *Ka/Ks =* 1.04, in “Metabolic pathways”, ko01100), according to annotated information. Then, we further manually confirmed the genes using online BLAST in NCBI. Furthermore, we detected expression profiles of the four target genes in the transcriptomic database of different normal tissues of *B. belcheri* (http://wcy.pkusulab.com/). *HMGCL* showed the highest expression in the skin, *SAT2* in the intestine, and *CYBS* and *MDH2* showed specific expression in muscle. Therefore, adult individuals were fed and treated to empty contents of intestine before sampling, following the methods of Zhang et al. [52]. Then, we collected three tissues (intestine, skin and muscle) of *B. belcheri* which were challenged with 1 mg/ml LPS according to the method of previous studies [53]. For each tissue, samples with three biological replications were collected at seven timing points after LPS challenge (0, 2, 6, 12, 24, 48 and 72 h; 10 μl/individual). Each sample group contained approximate 12 adult individuals. The samples collected at 0 h were only injected with PBS (used to dissolve LPS powder to 1 mg/ml) and used as the control. All samples were stored in TRIzol reagent (Invitrogen, Carlsbad, CA) at -20°C until use.

### RNA extraction and cDNA synthesis

Total RNA was isolated from each sample according to a previous protocol [54]. Residual genomic DNA was removed with an RNase-free DNase kit (Qiagen, Germany) following the manufacturer’s protocols. The RNA concentration and purity were determined by examining the OD_260_/D_280_ ratio with expected values between 1.8 and 2.0 using a BioPhotometer Plus (Eppendorf, Germany). Single-stranded synthesis was conducted using a RervertAid First Strand cDNA Synthesis kit (Thermo Scientific, USA). The cDNA (100 ng/μl) was diluted with RNase-free water for use in further analyses.

### Quantitative real-time PCR and statistical analyses

qRT-PCR was performed in a 20 μl reaction volume using the ABI 7300 real-time PCR system (Applied Biosystems, USA) with the SYBR Premix Ex Taq (Takara, Japan) according to manufacturer’s protocol. The reaction volume contained 1 μl (approximately 100 ng) of cDNA, 1 μl of each sense and anti-sense primer (10μM), 10 μl of 2 × SYBR Green Premix and 7 μl of ddH_2_O. The following PCR running program was used: 95 °C for 60 s, followed by 40 cycles of 95 °C for 10 s, 57 °C for 30 s and 72 °C for 30 s. The PCR reaction for each of three biological replicates was implemented according to the procedure described above, and each reaction was performed in triplicate. Melting curves were used to analyze the specificity of amplifications. Specific primers for all the genes used in this study were designed using Beacon Designer 7 (Supplementary Table 11), and the ribosomal protein S20 gene (*S20*) was used as the endogenous control [52]. The relative expression level of each gene was analyzed according to the 2^-ΔΔCT^method [55]. Data are presented as the means ± standard deviation (SD). Statistical analysis, one-way ANOVA, was performed using the IBM SPSS statistical software package 22.0, with significance declared at *P* < 0.05.

## SUPPLEMENTARY MATERIALS TABLES


