# Supplementary material for: Genome-wide comparison of the protein-coding repertoire reveals fast evolution of immune-related genes in cephalochordates and Osteichthyes superclass
Source: Oncotarget. 2017 Nov 28;9(1):83–95. doi: 10.18632/oncotarget.22749 (PMC5787515; doi:10.18632/oncotarget.22749)
Supplement: Supplementary file 1 [file oncotarget-09-83-s001.docx]

**Genome-wide comparison of the protein-coding repertoire reveals fast evolution of immune-related genes in cephalochordates and Osteichthyes superclass**

**Supplementary Material**

**Table S1. Statistics for annotation information of gene sets.**

|  | *Branchiostoma belcheri* | *B. floridae* | *Danio rerio* | *Latimeria chalumnae* | *Takifugu rubripes* |
| --- | --- | --- | --- | --- | --- |
| Total gene number | 30392 | 28627 | 26459 | 19569 | 18523 |
| Gene number with NR annotation | 27881 | 28602 | 25622 | 19471 | 18502 |
| Gene number with GO annotation | 16926 | 18229 | 10674 | 6121 | 7346 |
| Gene number with KEGG annotation | 19559 | 23039 | 20809 | 17986 | 17214 |

**Table S2. List of the enriched GO terms for particularly fast-evolving genes (identified by the top 5% *Ka*) in *Branchiostoma belcheri* vs. *B. floridae*.**

| Gene ontology | GO term id | GO term description | FDR (BH) | Flag (immune-related GO terms) |
| --- | --- | --- | --- | --- |
| cellular component | GO:0044424 | intracellular part | 7.66E-14 | # |
| cellular component | GO:0005622 | intracellular | 7.66E-14 | # |
| cellular component | GO:0043227 | membrane-bounded organelle | 3.61E-09 | # |
| cellular component | GO:0043226 | organelle | 3.61E-09 | # |
| cellular component | GO:0043229 | intracellular organelle | 3.93E-09 | # |
| cellular component | GO:0043231 | intracellular membrane-bounded organelle | 2.09E-08 | # |
| biological process | GO:0044237 | cellular metabolic process | 2.09E-08 | # |
| cellular component | GO:0005623 | cell | 3.47E-07 | # |
| cellular component | GO:0044464 | cell part | 3.47E-07 | # |
| biological process | GO:0023052 | signaling | 3.07E-06 |  |
| biological process | GO:0008152 | metabolic process | 3.29E-06 | # |
| biological process | GO:0071704 | organic substance metabolic process | 3.59E-06 |  |
| molecular function | GO:0004872 | receptor activity | 6.85E-06 | # |
| cellular component | GO:0030529 | ribonucleoprotein complex | 2.39E-05 | # |
| cellular component | GO:0044422 | organelle part | 3.24E-05 | # |
| biological process | GO:0007154 | cell communication | 5.50E-05 | # |
| cellular component | GO:0031967 | organelle envelope | 6.58E-05 | # |
| biological process | GO:0044238 | primary metabolic process | 6.58E-05 | # |
| cellular component | GO:0032991 | macromolecular complex | 6.72E-05 | # |
| molecular function | GO:0060089 | molecular transducer activity | 6.72E-05 | # |
| biological process | GO:0010467 | gene expression | 6.72E-05 |  |
| biological process | GO:0044700 | single organism signaling | 7.68E-05 |  |
| molecular function | GO:0022857 | transmembrane transporter activity | 1.32E-04 | # |
| molecular function | GO:0004871 | signal transducer activity | 1.69E-04 | # |
| biological process | GO:0007165 | signal transduction | 2.66E-04 | # |
| cellular component | GO:0031975 | envelope | 3.55E-04 | # |
| cellular component | GO:0044446 | intracellular organelle part | 5.21E-04 | # |
| molecular function | GO:0022891 | substrate-specific transmembrane transporter activity | 5.21E-04 | # |
| cellular component | GO:0019866 | organelle inner membrane | 5.89E-04 | # |
| molecular function | GO:0005198 | structural molecule activity | 7.66E-04 | # |
| cellular component | GO:0031090 | organelle membrane | 1.01E-03 | # |
| biological process | GO:0043170 | macromolecule metabolic process | 1.05E-03 | # |
| cellular component | GO:0044429 | mitochondrial part | 1.10E-03 | # |
| biological process | GO:0044249 | cellular biosynthetic process | 1.10E-03 | # |
| biological process | GO:0009058 | biosynthetic process | 1.12E-03 | # |
| biological process | GO:1901576 | organic substance biosynthetic process | 1.12E-03 |  |
| biological process | GO:0044260 | cellular macromolecule metabolic process | 1.17E-03 | # |
| cellular component | GO:0005739 | mitochondrion | 1.56E-03 | # |
| molecular function | GO:0005215 | transporter activity | 1.56E-03 | # |
| molecular function | GO:0022892 | substrate-specific transporter activity | 1.81E-03 | # |
| cellular component | GO:0005743 | mitochondrial inner membrane | 1.90E-03 | # |
| biological process | GO:0016071 | mRNA metabolic process | 1.90E-03 |  |
| biological process | GO:0016070 | RNA metabolic process | 2.34E-03 | # |
| cellular component | GO:0005634 | nucleus | 2.78E-03 | # |
| biological process | GO:0050896 | response to stimulus | 2.78E-03 | # |
| biological process | GO:0009059 | macromolecule biosynthetic process | 2.96E-03 | # |
| biological process | GO:0034645 | cellular macromolecule biosynthetic process | 3.70E-03 | # |
| biological process | GO:0006487 | protein N-linked glycosylation | 4.68E-03 |  |
| biological process | GO:0006807 | nitrogen compound metabolic process | 5.31E-03 | # |
| biological process | GO:0090304 | nucleic acid metabolic process | 5.63E-03 |  |
| biological process | GO:0006811 | ion transport | 6.63E-03 |  |
| biological process | GO:0006563 | L-serine metabolic process | 7.18E-03 |  |
| biological process | GO:0006396 | RNA processing | 8.05E-03 |  |
| biological process | GO:0005975 | carbohydrate metabolic process | 9.17E-03 | # |
| biological process | GO:0044723 | single-organism carbohydrate metabolic process | 9.81E-03 |  |
| biological process | GO:1901360 | organic cyclic compound metabolic process | 1.01E-02 |  |
| cellular component | GO:0044444 | cytoplasmic part | 1.05E-02 | # |
| molecular function | GO:0015002 | heme-copper terminal oxidase activity | 1.19E-02 |  |
| molecular function | GO:0048038 | quinone binding | 1.25E-02 |  |
| biological process | GO:0006725 | cellular aromatic compound metabolic process | 1.27E-02 | # |
| biological process | GO:0046483 | heterocycle metabolic process | 1.27E-02 | # |
| biological process | GO:0022414 | reproductive process | 1.27E-02 | # |
| biological process | GO:0007166 | cell surface receptor signaling pathway | 1.27E-02 | # |
| cellular component | GO:0005737 | cytoplasm | 1.52E-02 | # |
| cellular component | GO:0044428 | nuclear part | 1.52E-02 | # |
| biological process | GO:0034641 | cellular nitrogen compound metabolic process | 1.52E-02 | # |
| biological process | GO:0006091 | generation of precursor metabolites and energy | 1.52E-02 | # |
| molecular function | GO:0046872 | metal ion binding | 1.54E-02 | # |
| cellular component | GO:0005740 | mitochondrial envelope | 1.57E-02 | # |
| cellular component | GO:0031966 | mitochondrial membrane | 1.67E-02 | # |
| biological process | GO:0006139 | nucleobase-containing compound metabolic process | 1.74E-02 | # |
| biological process | GO:0051186 | cofactor metabolic process | 1.74E-02 |  |
| biological process | GO:0051716 | cellular response to stimulus | 2.24E-02 | # |
| biological process | GO:0001510 | RNA methylation | 2.59E-02 |  |
| biological process | GO:0002067 | glandular epithelial cell differentiation | 2.59E-02 |  |
| biological process | GO:0045333 | cellular respiration | 2.93E-02 |  |
| biological process | GO:0055114 | oxidation-reduction process | 3.01E-02 | # |
| cellular component | GO:0005840 | ribosome | 3.08E-02 | # |
| biological process | GO:0000003 | reproduction | 3.08E-02 | # |
| biological process | GO:0009069 | serine family amino acid metabolic process | 3.08E-02 |  |
| biological process | GO:0006486 | protein glycosylation | 3.08E-02 |  |
| biological process | GO:0043413 | macromolecule glycosylation | 3.08E-02 |  |
| biological process | GO:0070085 | glycosylation | 3.08E-02 |  |
| molecular function | GO:0016741 | transferase activity, transferring one-carbon groups | 3.12E-02 | # |
| biological process | GO:0016032 | viral reproduction | 3.12E-02 |  |
| biological process | GO:0022415 | viral reproductive process | 3.12E-02 | # |
| biological process | GO:0022900 | electron transport chain | 3.15E-02 | # |
| biological process | GO:0044764 | multi-organism cellular process | 3.34E-02 |  |
| biological process | GO:0046474 | glycerophospholipid biosynthetic process | 3.69E-02 |  |
| biological process | GO:0033036 | macromolecule localization | 3.86E-02 |  |
| molecular function | GO:0008175 | tRNA methyltransferase activity | 3.90E-02 |  |
| molecular function | GO:0016616 | oxidoreductase activity, acting on the CH-OH group of donors, NAD or NADP as acceptor | 4.07E-02 | # |
| molecular function | GO:0008168 | methyltransferase activity | 4.07E-02 | # |
| biological process | GO:0045017 | glycerolipid biosynthetic process | 4.07E-02 |  |
| biological process | GO:0008380 | RNA splicing | 4.07E-02 |  |
| biological process | GO:0015031 | protein transport | 4.07E-02 | # |
| biological process | GO:0050789 | regulation of biological process | 4.07E-02 | # |
| molecular function | GO:0043167 | ion binding | 4.08E-02 | # |
| molecular function | GO:0004540 | ribonuclease activity | 4.13E-02 |  |
| biological process | GO:0006006 | glucose metabolic process | 4.13E-02 | # |
| biological process | GO:0019318 | hexose metabolic process | 4.13E-02 | # |
| biological process | GO:0045184 | establishment of protein localization | 4.13E-02 | # |
| cellular component | GO:0000428 | DNA-directed RNA polymerase complex | 4.29E-02 | # |
| cellular component | GO:0016591 | DNA-directed RNA polymerase II, holoenzyme | 4.29E-02 | # |
| cellular component | GO:0030880 | RNA polymerase complex | 4.29E-02 | # |
| cellular component | GO:0055029 | nuclear DNA-directed RNA polymerase complex | 4.29E-02 | # |
| biological process | GO:0043414 | macromolecule methylation | 4.29E-02 |  |
| biological process | GO:0033013 | tetrapyrrole metabolic process | 4.29E-02 |  |
| molecular function | GO:0000030 | mannosyltransferase activity | 4.47E-02 |  |
| molecular function | GO:0016868 | intramolecular transferase activity, phosphotransferases | 4.47E-02 |  |
| molecular function | GO:0016835 | carbon-oxygen lyase activity | 4.58E-02 | # |
| biological process | GO:0019438 | aromatic compound biosynthetic process | 4.58E-02 |  |
| biological process | GO:0032259 | methylation | 4.60E-02 |  |
| biological process | GO:0019058 | viral infectious cycle | 4.67E-02 | # |
| biological process | GO:0008654 | phospholipid biosynthetic process | 4.70E-02 | # |
| molecular function | GO:0008757 | S-adenosylmethionine-dependent methyltransferase activity | 4.74E-02 | # |
| molecular function | GO:0043169 | cation binding | 4.74E-02 | # |
| biological process | GO:0009101 | glycoprotein biosynthetic process | 4.74E-02 | # |
| biological process | GO:0043623 | cellular protein complex assembly | 4.74E-02 | # |
| biological process | GO:0000377 | RNA splicing, via transesterification reactions with bulged adenosine as nucleophile | 4.74E-02 |  |
| biological process | GO:0005996 | monosaccharide metabolic process | 4.74E-02 | # |
| cellular component | GO:0031227 | intrinsic to endoplasmic reticulum membrane | 4.75E-02 | # |
| biological process | GO:1901362 | organic cyclic compound biosynthetic process | 4.75E-02 |  |
| biological process | GO:0044271 | cellular nitrogen compound biosynthetic process | 4.75E-02 | # |
| biological process | GO:0044703 | multi-organism reproductive process | 4.75E-02 |  |
| biological process | GO:0006397 | mRNA processing | 4.75E-02 |  |
| biological process | GO:0000375 | RNA splicing, via transesterification reactions | 4.75E-02 |  |
| molecular function | GO:0004518 | nuclease activity | 4.80E-02 |  |
| biological process | GO:0006351 | transcription, DNA-dependent | 4.80E-02 | # |
| biological process | GO:0032774 | RNA biosynthetic process | 4.80E-02 | # |

**Table S3. List of the enriched GO terms for particularly fast-evolving genes (identified by the top 5% *Ka/Ks*) in *Branchiostoma belcheri* vs. *B. floridae*.**

| Gene ontology | GO term id | GO term description | FDR (BH) | Flag (immune-related GO terms) |
| --- | --- | --- | --- | --- |
| biological process | GO:0044237 | cellular metabolic process | 2.68E-08 | # |
| biological process | GO:0071704 | organic substance metabolic process | 3.50E-06 |  |
| biological process | GO:0008152 | metabolic process | 3.78E-06 | # |
| biological process | GO:0023052 | signaling | 6.31E-06 |  |
| biological process | GO:0010467 | gene expression | 4.93E-05 |  |
| biological process | GO:0044238 | primary metabolic process | 6.33E-05 | # |
| biological process | GO:0007154 | cell communication | 1.04E-04 | # |
| biological process | GO:0044700 | single organism signaling | 1.75E-04 |  |
| biological process | GO:0007165 | signal transduction | 1.98E-04 | # |
| biological process | GO:0043170 | macromolecule metabolic process | 3.11E-04 | # |
| biological process | GO:0044260 | cellular macromolecule metabolic process | 3.41E-04 | # |
| biological process | GO:0016070 | RNA metabolic process | 7.24E-04 | # |
| biological process | GO:0044249 | cellular biosynthetic process | 1.20E-03 | # |
| biological process | GO:0009058 | biosynthetic process | 1.20E-03 | # |
| biological process | GO:1901576 | organic substance biosynthetic process | 1.22E-03 |  |
| biological process | GO:0090304 | nucleic acid metabolic process | 1.34E-03 |  |
| biological process | GO:0016071 | mRNA metabolic process | 1.96E-03 |  |
| biological process | GO:0009059 | macromolecule biosynthetic process | 3.26E-03 | # |
| biological process | GO:0034645 | cellular macromolecule biosynthetic process | 3.99E-03 | # |
| biological process | GO:0006807 | nitrogen compound metabolic process | 4.15E-03 | # |
| biological process | GO:1901360 | organic cyclic compound metabolic process | 4.54E-03 |  |
| biological process | GO:0006487 | protein N-linked glycosylation | 4.63E-03 |  |
| biological process | GO:0006725 | cellular aromatic compound metabolic process | 5.87E-03 | # |
| biological process | GO:0050896 | response to stimulus | 5.87E-03 | # |
| biological process | GO:0046483 | heterocycle metabolic process | 5.91E-03 | # |
| biological process | GO:0006811 | ion transport | 6.18E-03 |  |
| biological process | GO:0006563 | L-serine metabolic process | 6.77E-03 |  |
| biological process | GO:0034641 | cellular nitrogen compound metabolic process | 7.13E-03 | # |
| biological process | GO:0006396 | RNA processing | 7.89E-03 |  |
| biological process | GO:0006139 | nucleobase-containing compound metabolic process | 8.43E-03 | # |
| biological process | GO:0005975 | carbohydrate metabolic process | 8.88E-03 | # |
| biological process | GO:0044723 | single-organism carbohydrate metabolic process | 9.10E-03 |  |
| biological process | GO:0007166 | cell surface receptor signaling pathway | 9.10E-03 | # |
| biological process | GO:0006091 | generation of precursor metabolites and energy | 1.56E-02 | # |
| biological process | GO:0033036 | macromolecule localization | 1.71E-02 |  |
| biological process | GO:0043623 | cellular protein complex assembly | 1.81E-02 | # |
| biological process | GO:0051186 | cofactor metabolic process | 1.81E-02 |  |
| biological process | GO:0015031 | protein transport | 2.04E-02 | # |
| biological process | GO:0045184 | establishment of protein localization | 2.16E-02 | # |
| biological process | GO:0001510 | RNA methylation | 2.49E-02 |  |
| biological process | GO:0002067 | glandular epithelial cell differentiation | 2.49E-02 |  |
| biological process | GO:0045333 | cellular respiration | 2.80E-02 |  |
| biological process | GO:0055114 | oxidation-reduction process | 2.93E-02 | # |
| biological process | GO:0009069 | serine family amino acid metabolic process | 2.95E-02 |  |
| biological process | GO:0006486 | protein glycosylation | 2.98E-02 |  |
| biological process | GO:0043413 | macromolecule glycosylation | 2.98E-02 |  |
| biological process | GO:0070085 | glycosylation | 2.98E-02 |  |
| biological process | GO:0016032 | viral reproduction | 3.04E-02 |  |
| biological process | GO:0022415 | viral reproductive process | 3.04E-02 | # |
| biological process | GO:0022900 | electron transport chain | 3.04E-02 | # |
| biological process | GO:0051716 | cellular response to stimulus | 3.04E-02 | # |
| biological process | GO:0044764 | multi-organism cellular process | 3.16E-02 |  |
| biological process | GO:0046474 | glycerophospholipid biosynthetic process | 3.42E-02 |  |
| biological process | GO:0071702 | organic substance transport | 3.54E-02 |  |
| biological process | GO:0022414 | reproductive process | 3.60E-02 | # |
| biological process | GO:0045017 | glycerolipid biosynthetic process | 3.85E-02 |  |
| biological process | GO:0008380 | RNA splicing | 3.89E-02 |  |
| biological process | GO:0006006 | glucose metabolic process | 3.98E-02 | # |
| biological process | GO:0019318 | hexose metabolic process | 4.00E-02 | # |
| biological process | GO:0043414 | macromolecule methylation | 4.07E-02 |  |
| biological process | GO:0033013 | tetrapyrrole metabolic process | 4.12E-02 |  |
| biological process | GO:0000003 | reproduction | 4.30E-02 | # |
| biological process | GO:0019438 | aromatic compound biosynthetic process | 4.43E-02 |  |
| biological process | GO:0032259 | methylation | 4.43E-02 |  |
| biological process | GO:0019058 | viral infectious cycle | 4.45E-02 | # |
| biological process | GO:0008654 | phospholipid biosynthetic process | 4.47E-02 | # |
| biological process | GO:0009101 | glycoprotein biosynthetic process | 4.63E-02 | # |
| biological process | GO:0000377 | RNA splicing, via transesterification reactions with bulged adenosine as nucleophile | 4.63E-02 |  |
| biological process | GO:0005996 | monosaccharide metabolic process | 4.63E-02 | # |
| biological process | GO:1901362 | organic cyclic compound biosynthetic process | 4.66E-02 |  |
| biological process | GO:0044271 | cellular nitrogen compound biosynthetic process | 4.68E-02 | # |
| biological process | GO:0044703 | multi-organism reproductive process | 4.68E-02 |  |
| biological process | GO:0006397 | mRNA processing | 4.68E-02 |  |
| biological process | GO:0000375 | RNA splicing, via transesterification reactions | 4.69E-02 |  |
| biological process | GO:0006351 | transcription, DNA-dependent | 4.80E-02 | # |
| biological process | GO:0032774 | RNA biosynthetic process | 4.82E-02 | # |
| biological process | GO:0034622 | cellular macromolecular complex assembly | 4.82E-02 | # |
| cellular component | GO:0044424 | intracellular part | 7.74E-14 | # |
| cellular component | GO:0005622 | intracellular | 7.74E-14 | # |
| cellular component | GO:0043226 | organelle | 4.27E-09 | # |
| cellular component | GO:0043229 | intracellular organelle | 4.96E-09 | # |
| cellular component | GO:0043227 | membrane-bounded organelle | 7.60E-09 | # |
| cellular component | GO:0043231 | intracellular membrane-bounded organelle | 4.71E-08 | # |
| cellular component | GO:0005623 | cell | 1.32E-07 | # |
| cellular component | GO:0044464 | cell part | 1.32E-07 | # |
| cellular component | GO:0030529 | ribonucleoprotein complex | 6.31E-06 | # |
| cellular component | GO:0032991 | macromolecular complex | 3.46E-05 | # |
| cellular component | GO:0044422 | organelle part | 6.21E-05 | # |
| cellular component | GO:0031967 | organelle envelope | 6.31E-05 | # |
| cellular component | GO:0031975 | envelope | 3.41E-04 | # |
| cellular component | GO:0019866 | organelle inner membrane | 5.76E-04 | # |
| cellular component | GO:0044429 | mitochondrial part | 1.11E-03 | # |
| cellular component | GO:0044446 | intracellular organelle part | 1.23E-03 | # |
| cellular component | GO:0005739 | mitochondrion | 1.52E-03 | # |
| cellular component | GO:0031090 | organelle membrane | 1.52E-03 | # |
| cellular component | GO:0005743 | mitochondrial inner membrane | 1.85E-03 | # |
| cellular component | GO:0005634 | nucleus | 2.78E-03 | # |
| cellular component | GO:0044444 | cytoplasmic part | 5.91E-03 | # |
| cellular component | GO:0005737 | cytoplasm | 9.09E-03 | # |
| cellular component | GO:0044428 | nuclear part | 1.53E-02 | # |
| cellular component | GO:0005740 | mitochondrial envelope | 1.58E-02 | # |
| cellular component | GO:0031966 | mitochondrial membrane | 1.67E-02 | # |
| cellular component | GO:0031224 | intrinsic to membrane | 2.31E-02 | # |
| cellular component | GO:0005840 | ribosome | 2.98E-02 | # |
| cellular component | GO:0016020 | membrane | 2.98E-02 | # |
| cellular component | GO:0044425 | membrane part | 3.23E-02 | # |
| cellular component | GO:0043234 | protein complex | 3.55E-02 | # |
| cellular component | GO:0000428 | DNA-directed RNA polymerase complex | 4.07E-02 | # |
| cellular component | GO:0016591 | DNA-directed RNA polymerase II, holoenzyme | 4.07E-02 | # |
| cellular component | GO:0030880 | RNA polymerase complex | 4.07E-02 | # |
| cellular component | GO:0055029 | nuclear DNA-directed RNA polymerase complex | 4.07E-02 | # |
| cellular component | GO:0031227 | intrinsic to endoplasmic reticulum membrane | 4.63E-02 | # |
| molecular function | GO:0004872 | receptor activity | 6.53E-06 | # |
| molecular function | GO:0060089 | molecular transducer activity | 5.50E-05 | # |
| molecular function | GO:0022857 | transmembrane transporter activity | 1.42E-04 | # |
| molecular function | GO:0004871 | signal transducer activity | 1.75E-04 | # |
| molecular function | GO:0022891 | substrate-specific transmembrane transporter activity | 5.09E-04 | # |
| molecular function | GO:0005198 | structural molecule activity | 7.82E-04 | # |
| molecular function | GO:0005215 | transporter activity | 1.20E-03 | # |
| molecular function | GO:0022892 | substrate-specific transporter activity | 1.77E-03 | # |
| molecular function | GO:0046872 | metal ion binding | 4.54E-03 | # |
| molecular function | GO:0015002 | heme-copper terminal oxidase activity | 1.10E-02 |  |
| molecular function | GO:0048038 | quinone binding | 1.15E-02 |  |
| molecular function | GO:0019901 | protein kinase binding | 1.62E-02 | # |
| molecular function | GO:0004518 | nuclease activity | 2.26E-02 |  |
| molecular function | GO:0043167 | ion binding | 2.62E-02 | # |
| molecular function | GO:0051540 | metal cluster binding | 2.63E-02 |  |
| molecular function | GO:0016741 | transferase activity, transferring one-carbon groups | 3.04E-02 | # |
| molecular function | GO:0043169 | cation binding | 3.16E-02 | # |
| molecular function | GO:0008175 | tRNA methyltransferase activity | 3.57E-02 |  |
| molecular function | GO:0016616 | oxidoreductase activity, acting on the CH-OH group of donors, NAD or NADP as acceptor | 3.85E-02 | # |
| molecular function | GO:0008168 | methyltransferase activity | 3.89E-02 | # |
| molecular function | GO:0004540 | ribonuclease activity | 3.98E-02 |  |
| molecular function | GO:0000030 | mannosyltransferase activity | 4.23E-02 |  |
| molecular function | GO:0016868 | intramolecular transferase activity, phosphotransferases | 4.23E-02 |  |
| molecular function | GO:0046914 | transition metal ion binding | 4.23E-02 |  |
| molecular function | GO:0016835 | carbon-oxygen lyase activity | 4.39E-02 | # |
| molecular function | GO:0008757 | S-adenosylmethionine-dependent methyltransferase activity | 4.59E-02 | # |

**Table S4. List of the enriched GO terms for moderately fast-evolving genes (identified by the top 10% *Ka*) in *Branchiostoma belcheri* vs. *B. floridae*.**

| Gene ontology | GO term id | GO term description | FDR (BH) | Flag (immune-related GO terms) |
| --- | --- | --- | --- | --- |
| biological process | GO:0071704 | organic substance metabolic process | 3.00E-14 |  |
| biological process | GO:0044237 | cellular metabolic process | 3.00E-14 | # |
| biological process | GO:0008152 | metabolic process | 3.86E-14 | # |
| biological process | GO:0044238 | primary metabolic process | 2.75E-12 | # |
| biological process | GO:0043170 | macromolecule metabolic process | 2.81E-10 | # |
| biological process | GO:0010467 | gene expression | 3.37E-09 |  |
| biological process | GO:0023052 | signaling | 2.81E-08 |  |
| biological process | GO:0006807 | nitrogen compound metabolic process | 3.54E-07 | # |
| biological process | GO:1901576 | organic substance biosynthetic process | 1.03E-06 |  |
| biological process | GO:0044260 | cellular macromolecule metabolic process | 2.00E-06 | # |
| biological process | GO:0009058 | biosynthetic process | 2.97E-06 | # |
| biological process | GO:0006725 | cellular aromatic compound metabolic process | 4.70E-06 | # |
| biological process | GO:1901360 | organic cyclic compound metabolic process | 4.84E-06 |  |
| biological process | GO:0046483 | heterocycle metabolic process | 6.23E-06 | # |
| biological process | GO:0044249 | cellular biosynthetic process | 6.53E-06 | # |
| biological process | GO:0034641 | cellular nitrogen compound metabolic process | 7.56E-06 | # |
| biological process | GO:0006139 | nucleobase-containing compound metabolic process | 8.68E-06 | # |
| biological process | GO:0045184 | establishment of protein localization | 9.09E-06 | # |
| biological process | GO:0033036 | macromolecule localization | 1.21E-05 |  |
| biological process | GO:0015031 | protein transport | 2.05E-05 | # |
| biological process | GO:0008104 | protein localization | 2.60E-05 |  |
| biological process | GO:0044710 | single-organism metabolic process | 3.05E-05 |  |
| biological process | GO:0034645 | cellular macromolecule biosynthetic process | 3.82E-05 | # |
| biological process | GO:0009059 | macromolecule biosynthetic process | 3.82E-05 | # |
| biological process | GO:0046907 | intracellular transport | 4.40E-05 | # |
| biological process | GO:0016070 | RNA metabolic process | 4.40E-05 | # |
| biological process | GO:0007166 | cell surface receptor signaling pathway | 5.97E-05 | # |
| biological process | GO:0090304 | nucleic acid metabolic process | 7.40E-05 |  |
| biological process | GO:0051649 | establishment of localization in cell | 8.20E-05 | # |
| biological process | GO:0007154 | cell communication | 1.10E-04 | # |
| biological process | GO:0006396 | RNA processing | 1.53E-04 |  |
| biological process | GO:0044700 | single organism signaling | 1.53E-04 |  |
| biological process | GO:0071702 | organic substance transport | 1.95E-04 |  |
| biological process | GO:0051641 | cellular localization | 3.45E-04 | # |
| biological process | GO:0007165 | signal transduction | 3.54E-04 | # |
| biological process | GO:0016071 | mRNA metabolic process | 4.61E-04 |  |
| biological process | GO:0007186 | G-protein coupled receptor signaling pathway | 7.55E-04 | # |
| biological process | GO:0048193 | Golgi vesicle transport | 9.82E-04 |  |
| biological process | GO:0044281 | small molecule metabolic process | 1.05E-03 |  |
| biological process | GO:1901362 | organic cyclic compound biosynthetic process | 1.25E-03 |  |
| biological process | GO:0044271 | cellular nitrogen compound biosynthetic process | 1.31E-03 | # |
| biological process | GO:1901575 | organic substance catabolic process | 1.74E-03 |  |
| biological process | GO:0019438 | aromatic compound biosynthetic process | 1.74E-03 |  |
| biological process | GO:0018130 | heterocycle biosynthetic process | 1.86E-03 | # |
| biological process | GO:0006006 | glucose metabolic process | 1.97E-03 | # |
| biological process | GO:0006091 | generation of precursor metabolites and energy | 1.99E-03 | # |
| biological process | GO:0016192 | vesicle-mediated transport | 2.87E-03 | # |
| biological process | GO:0034654 | nucleobase-containing compound biosynthetic process | 3.86E-03 | # |
| biological process | GO:0051173 | positive regulation of nitrogen compound metabolic process | 4.50E-03 | # |
| biological process | GO:0019318 | hexose metabolic process | 4.63E-03 | # |
| biological process | GO:0009057 | macromolecule catabolic process | 4.82E-03 | # |
| biological process | GO:0009893 | positive regulation of metabolic process | 5.01E-03 | # |
| biological process | GO:0019538 | protein metabolic process | 5.25E-03 | # |
| biological process | GO:0008380 | RNA splicing | 5.58E-03 |  |
| biological process | GO:0009056 | catabolic process | 5.58E-03 | # |
| biological process | GO:0031325 | positive regulation of cellular metabolic process | 5.61E-03 | # |
| biological process | GO:0009987 | cellular process | 6.52E-03 | # |
| biological process | GO:0006563 | L-serine metabolic process | 6.64E-03 |  |
| biological process | GO:0006520 | cellular amino acid metabolic process | 7.41E-03 | # |
| biological process | GO:0016093 | polyprenol metabolic process | 7.60E-03 |  |
| biological process | GO:0000377 | RNA splicing, via transesterification reactions with bulged adenosine as nucleophile | 7.60E-03 |  |
| biological process | GO:0005996 | monosaccharide metabolic process | 7.60E-03 | # |
| biological process | GO:0000375 | RNA splicing, via transesterification reactions | 8.28E-03 |  |
| biological process | GO:0031323 | regulation of cellular metabolic process | 8.98E-03 | # |
| biological process | GO:0045333 | cellular respiration | 9.08E-03 |  |
| biological process | GO:0044265 | cellular macromolecule catabolic process | 9.09E-03 | # |
| biological process | GO:0044723 | single-organism carbohydrate metabolic process | 9.55E-03 |  |
| biological process | GO:0055114 | oxidation-reduction process | 1.03E-02 | # |
| biological process | GO:0022900 | electron transport chain | 1.07E-02 | # |
| biological process | GO:0006886 | intracellular protein transport | 1.09E-02 | # |
| biological process | GO:0044085 | cellular component biogenesis | 1.09E-02 |  |
| biological process | GO:0016032 | viral reproduction | 1.13E-02 |  |
| biological process | GO:0022415 | viral reproductive process | 1.13E-02 | # |
| biological process | GO:0006007 | glucose catabolic process | 1.27E-02 |  |
| biological process | GO:0044764 | multi-organism cellular process | 1.29E-02 |  |
| biological process | GO:1901564 | organonitrogen compound metabolic process | 1.41E-02 |  |
| biological process | GO:0010257 | NADH dehydrogenase complex assembly | 1.43E-02 |  |
| biological process | GO:0019320 | hexose catabolic process | 1.47E-02 |  |
| biological process | GO:0006397 | mRNA processing | 1.51E-02 |  |
| biological process | GO:0006351 | transcription, DNA-dependent | 1.69E-02 | # |
| biological process | GO:0032774 | RNA biosynthetic process | 1.71E-02 | # |
| biological process | GO:0046365 | monosaccharide catabolic process | 1.71E-02 |  |
| biological process | GO:0044267 | cellular protein metabolic process | 1.93E-02 | # |
| biological process | GO:0000956 | nuclear-transcribed mRNA catabolic process | 1.95E-02 |  |
| biological process | GO:0006352 | DNA-dependent transcription, initiation | 1.95E-02 | # |
| biological process | GO:0006487 | protein N-linked glycosylation | 1.95E-02 |  |
| biological process | GO:0044248 | cellular catabolic process | 2.03E-02 | # |
| biological process | GO:0006402 | mRNA catabolic process | 2.19E-02 |  |
| biological process | GO:0005975 | carbohydrate metabolic process | 2.19E-02 | # |
| biological process | GO:0006013 | mannose metabolic process | 2.41E-02 |  |
| biological process | GO:0009139 | pyrimidine nucleoside diphosphate biosynthetic process | 2.41E-02 |  |
| biological process | GO:0009186 | deoxyribonucleoside diphosphate metabolic process | 2.41E-02 |  |
| biological process | GO:0009189 | deoxyribonucleoside diphosphate biosynthetic process | 2.41E-02 |  |
| biological process | GO:0009196 | pyrimidine deoxyribonucleoside diphosphate metabolic process | 2.41E-02 |  |
| biological process | GO:0009197 | pyrimidine deoxyribonucleoside diphosphate biosynthetic process | 2.41E-02 |  |
| biological process | GO:0046500 | S-adenosylmethionine metabolic process | 2.41E-02 |  |
| biological process | GO:0046677 | response to antibiotic | 2.41E-02 | # |
| biological process | GO:0050680 | negative regulation of epithelial cell proliferation | 2.41E-02 |  |
| biological process | GO:0006289 | nucleotide-excision repair | 2.45E-02 | # |
| biological process | GO:0019058 | viral infectious cycle | 2.45E-02 | # |
| biological process | GO:0071840 | cellular component organization or biogenesis | 2.48E-02 |  |
| biological process | GO:0006605 | protein targeting | 2.49E-02 |  |
| biological process | GO:0006508 | proteolysis | 2.49E-02 | # |
| biological process | GO:0009896 | positive regulation of catabolic process | 2.74E-02 | # |
| biological process | GO:0019080 | viral genome expression | 2.74E-02 |  |
| biological process | GO:0045935 | positive regulation of nucleobase-containing compound metabolic process | 2.74E-02 | # |
| biological process | GO:0051716 | cellular response to stimulus | 2.91E-02 | # |
| biological process | GO:0006412 | translation | 2.93E-02 | # |
| biological process | GO:0016052 | carbohydrate catabolic process | 3.05E-02 |  |
| biological process | GO:0044724 | single-organism carbohydrate catabolic process | 3.05E-02 |  |
| biological process | GO:0044703 | multi-organism reproductive process | 3.05E-02 |  |
| biological process | GO:0031016 | pancreas development | 3.13E-02 |  |
| biological process | GO:0051186 | cofactor metabolic process | 3.33E-02 |  |
| biological process | GO:0034613 | cellular protein localization | 3.36E-02 |  |
| biological process | GO:0006401 | RNA catabolic process | 3.42E-02 |  |
| biological process | GO:0051234 | establishment of localization | 3.42E-02 | # |
| biological process | GO:0070727 | cellular macromolecule localization | 3.56E-02 |  |
| biological process | GO:0022402 | cell cycle process | 4.12E-02 | # |
| biological process | GO:0019941 | modification-dependent protein catabolic process | 4.25E-02 | # |
| biological process | GO:0043632 | modification-dependent macromolecule catabolic process | 4.25E-02 | # |
| biological process | GO:0080090 | regulation of primary metabolic process | 4.67E-02 |  |
| biological process | GO:0051603 | proteolysis involved in cellular protein catabolic process | 4.71E-02 | # |
| biological process | GO:0006066 | alcohol metabolic process | 4.73E-02 | # |
| biological process | GO:0006473 | protein acetylation | 4.79E-02 |  |
| biological process | GO:0044257 | cellular protein catabolic process | 4.83E-02 | # |
| biological process | GO:0006082 | organic acid metabolic process | 4.87E-02 | # |
| biological process | GO:0006974 | response to DNA damage stimulus | 4.97E-02 | # |
| cellular component | GO:0044424 | intracellular part | 3.00E-14 | # |
| cellular component | GO:0005622 | intracellular | 3.00E-14 | # |
| cellular component | GO:0043226 | organelle | 3.00E-14 | # |
| cellular component | GO:0043229 | intracellular organelle | 3.00E-14 | # |
| cellular component | GO:0043227 | membrane-bounded organelle | 3.00E-14 | # |
| cellular component | GO:0043231 | intracellular membrane-bounded organelle | 3.00E-14 | # |
| cellular component | GO:0005623 | cell | 3.00E-14 | # |
| cellular component | GO:0044464 | cell part | 3.00E-14 | # |
| cellular component | GO:0032991 | macromolecular complex | 3.00E-14 | # |
| cellular component | GO:0044422 | organelle part | 3.49E-14 | # |
| cellular component | GO:0044444 | cytoplasmic part | 3.73E-12 | # |
| cellular component | GO:0005737 | cytoplasm | 1.79E-11 | # |
| cellular component | GO:0044446 | intracellular organelle part | 2.78E-11 | # |
| cellular component | GO:0030529 | ribonucleoprotein complex | 1.19E-09 | # |
| cellular component | GO:0005634 | nucleus | 2.81E-08 | # |
| cellular component | GO:0031090 | organelle membrane | 1.89E-07 | # |
| cellular component | GO:0044428 | nuclear part | 3.81E-07 | # |
| cellular component | GO:0031967 | organelle envelope | 6.34E-07 | # |
| cellular component | GO:0043233 | organelle lumen | 3.18E-06 | # |
| cellular component | GO:0070013 | intracellular organelle lumen | 3.18E-06 | # |
| cellular component | GO:0031981 | nuclear lumen | 4.70E-06 | # |
| cellular component | GO:0043234 | protein complex | 4.70E-06 | # |
| cellular component | GO:0031974 | membrane-enclosed lumen | 4.84E-06 | # |
| cellular component | GO:0044429 | mitochondrial part | 6.28E-06 | # |
| cellular component | GO:0019866 | organelle inner membrane | 1.00E-05 | # |
| cellular component | GO:0005743 | mitochondrial inner membrane | 1.47E-05 | # |
| cellular component | GO:0005739 | mitochondrion | 2.15E-05 | # |
| cellular component | GO:0031975 | envelope | 2.24E-05 | # |
| cellular component | GO:0031966 | mitochondrial membrane | 3.50E-05 | # |
| cellular component | GO:0005740 | mitochondrial envelope | 5.79E-05 | # |
| cellular component | GO:0031224 | intrinsic to membrane | 7.55E-04 | # |
| cellular component | GO:0000502 | proteasome complex | 3.78E-03 | # |
| cellular component | GO:0012505 | endomembrane system | 3.86E-03 | # |
| cellular component | GO:0044451 | nucleoplasm part | 7.41E-03 | # |
| cellular component | GO:0030137 | COPI-coated vesicle | 7.89E-03 | # |
| cellular component | GO:0005654 | nucleoplasm | 8.74E-03 | # |
| cellular component | GO:0044391 | ribosomal subunit | 9.88E-03 |  |
| cellular component | GO:0005840 | ribosome | 1.33E-02 | # |
| cellular component | GO:0044425 | membrane part | 1.33E-02 | # |
| cellular component | GO:0016020 | membrane | 1.64E-02 | # |
| cellular component | GO:0070469 | respiratory chain | 1.64E-02 |  |
| cellular component | GO:0071944 | cell periphery | 1.84E-02 |  |
| cellular component | GO:0000428 | DNA-directed RNA polymerase complex | 2.19E-02 | # |
| cellular component | GO:0016591 | DNA-directed RNA polymerase II, holoenzyme | 2.19E-02 | # |
| cellular component | GO:0030880 | RNA polymerase complex | 2.19E-02 | # |
| cellular component | GO:0055029 | nuclear DNA-directed RNA polymerase complex | 2.19E-02 | # |
| cellular component | GO:0005886 | plasma membrane | 2.19E-02 | # |
| cellular component | GO:0031201 | SNARE complex | 2.45E-02 | # |
| cellular component | GO:0015934 | large ribosomal subunit | 2.52E-02 | # |
| cellular component | GO:0031227 | intrinsic to endoplasmic reticulum membrane | 2.95E-02 | # |
| cellular component | GO:0005681 | spliceosomal complex | 2.95E-02 | # |
| cellular component | GO:0005746 | mitochondrial respiratory chain | 3.05E-02 | # |
| cellular component | GO:0044455 | mitochondrial membrane part | 3.34E-02 | # |
| cellular component | GO:0044421 | extracellular region part | 3.36E-02 | # |
| cellular component | GO:0005839 | proteasome core complex | 3.47E-02 | # |
| cellular component | GO:0005576 | extracellular region | 4.25E-02 | # |
| cellular component | GO:0012506 | vesicle membrane | 4.73E-02 | # |
| cellular component | GO:0005789 | endoplasmic reticulum membrane | 4.92E-02 | # |
| molecular function | GO:0004872 | receptor activity | 2.32E-13 | # |
| molecular function | GO:0004888 | transmembrane signaling receptor activity | 4.64E-11 | # |
| molecular function | GO:0038023 | signaling receptor activity | 5.77E-11 |  |
| molecular function | GO:0004871 | signal transducer activity | 8.65E-08 | # |
| molecular function | GO:0060089 | molecular transducer activity | 1.73E-06 | # |
| molecular function | GO:0005198 | structural molecule activity | 3.17E-05 | # |
| molecular function | GO:0004661 | protein geranylgeranyltransferase activity | 4.85E-04 |  |
| molecular function | GO:0016765 | transferase activity, transferring alkyl or aryl (other than methyl) groups | 8.86E-04 | # |
| molecular function | GO:0004518 | nuclease activity | 1.41E-03 |  |
| molecular function | GO:0005216 | ion channel activity | 1.61E-03 | # |
| molecular function | GO:0022838 | substrate-specific channel activity | 1.61E-03 | # |
| molecular function | GO:0015267 | channel activity | 1.61E-03 | # |
| molecular function | GO:0022803 | passive transmembrane transporter activity | 1.61E-03 | # |
| molecular function | GO:0003676 | nucleic acid binding | 2.35E-03 | # |
| molecular function | GO:0008318 | protein prenyltransferase activity | 2.46E-03 |  |
| molecular function | GO:0008565 | protein transporter activity | 2.46E-03 |  |
| molecular function | GO:0003824 | catalytic activity | 2.75E-03 | # |
| molecular function | GO:0022836 | gated channel activity | 3.86E-03 | # |
| molecular function | GO:0097159 | organic cyclic compound binding | 4.32E-03 |  |
| molecular function | GO:0016853 | isomerase activity | 4.47E-03 | # |
| molecular function | GO:0022839 | ion gated channel activity | 5.25E-03 | # |
| molecular function | GO:0004527 | exonuclease activity | 5.58E-03 |  |
| molecular function | GO:1901363 | heterocyclic compound binding | 5.58E-03 |  |
| molecular function | GO:0016859 | cis-trans isomerase activity | 9.09E-03 |  |
| molecular function | GO:0015075 | ion transmembrane transporter activity | 9.58E-03 | # |
| molecular function | GO:0004519 | endonuclease activity | 1.06E-02 |  |
| molecular function | GO:0008320 | protein transmembrane transporter activity | 1.07E-02 |  |
| molecular function | GO:0022884 | macromolecule transmembrane transporter activity | 1.07E-02 |  |
| molecular function | GO:0008408 | 3'-5' exonuclease activity | 1.31E-02 |  |
| molecular function | GO:0004659 | prenyltransferase activity | 2.23E-02 |  |
| molecular function | GO:0005515 | protein binding | 2.39E-02 | # |
| molecular function | GO:0016835 | carbon-oxygen lyase activity | 2.44E-02 | # |
| molecular function | GO:0000030 | mannosyltransferase activity | 2.52E-02 |  |
| molecular function | GO:0008757 | S-adenosylmethionine-dependent methyltransferase activity | 2.67E-02 | # |
| molecular function | GO:0003677 | DNA binding | 3.05E-02 | # |
| molecular function | GO:0016741 | transferase activity, transferring one-carbon groups | 3.20E-02 | # |
| molecular function | GO:0022891 | substrate-specific transmembrane transporter activity | 3.22E-02 | # |
| molecular function | GO:0016861 | intramolecular oxidoreductase activity, interconverting aldoses and ketoses | 3.42E-02 |  |
| molecular function | GO:0022857 | transmembrane transporter activity | 3.42E-02 | # |
| molecular function | GO:0016802 | trialkylsulfonium hydrolase activity | 4.25E-02 |  |
| molecular function | GO:0048038 | quinone binding | 4.25E-02 |  |
| molecular function | GO:0070567 | cytidylyltransferase activity | 4.25E-02 |  |

**Table S5.** **List of the enriched GO terms for moderately fast-evolving genes (identified by the top 15% *Ka/Ks*) in *Branchiostoma belcheri* vs. *B. floridae*.**

| Gene ontology | GO term id | GO term description | FDR (BH) | Flag (immune-related GO terms) |
| --- | --- | --- | --- | --- |
| biological process | GO:0071704 | organic substance metabolic process | 4.72E-11 |  |
| biological process | GO:0044237 | cellular metabolic process | 1.59E-09 | # |
| biological process | GO:0008152 | metabolic process | 1.30E-08 | # |
| biological process | GO:0044238 | primary metabolic process | 9.08E-08 | # |
| biological process | GO:0010467 | gene expression | 8.23E-07 |  |
| biological process | GO:0043170 | macromolecule metabolic process | 3.13E-06 | # |
| biological process | GO:1901576 | organic substance biosynthetic process | 2.80E-05 |  |
| biological process | GO:0009058 | biosynthetic process | 6.53E-05 | # |
| biological process | GO:0044249 | cellular biosynthetic process | 1.13E-04 | # |
| biological process | GO:0006807 | nitrogen compound metabolic process | 1.13E-04 | # |
| biological process | GO:0023052 | signaling | 1.25E-04 |  |
| biological process | GO:1901360 | organic cyclic compound metabolic process | 3.34E-04 |  |
| biological process | GO:0044710 | single-organism metabolic process | 4.11E-04 |  |
| biological process | GO:0006725 | cellular aromatic compound metabolic process | 4.17E-04 | # |
| biological process | GO:0046483 | heterocycle metabolic process | 5.39E-04 | # |
| biological process | GO:0009059 | macromolecule biosynthetic process | 7.16E-04 | # |
| biological process | GO:0034645 | cellular macromolecule biosynthetic process | 7.44E-04 | # |
| biological process | GO:0006139 | nucleobase-containing compound metabolic process | 7.60E-04 | # |
| biological process | GO:0044260 | cellular macromolecule metabolic process | 7.60E-04 | # |
| biological process | GO:0034641 | cellular nitrogen compound metabolic process | 9.05E-04 | # |
| biological process | GO:0031325 | positive regulation of cellular metabolic process | 2.34E-03 | # |
| biological process | GO:0006396 | RNA processing | 3.37E-03 |  |
| biological process | GO:0007166 | cell surface receptor signaling pathway | 3.37E-03 | # |
| biological process | GO:0009893 | positive regulation of metabolic process | 3.58E-03 | # |
| biological process | GO:0007154 | cell communication | 3.67E-03 | # |
| biological process | GO:0016070 | RNA metabolic process | 3.72E-03 | # |
| biological process | GO:1901362 | organic cyclic compound biosynthetic process | 4.14E-03 |  |
| biological process | GO:0044271 | cellular nitrogen compound biosynthetic process | 4.31E-03 | # |
| biological process | GO:0016071 | mRNA metabolic process | 4.38E-03 |  |
| biological process | GO:0044700 | single organism signaling | 4.39E-03 |  |
| biological process | GO:0033036 | macromolecule localization | 4.56E-03 |  |
| biological process | GO:0044281 | small molecule metabolic process | 5.15E-03 |  |
| biological process | GO:0042278 | purine nucleoside metabolic process | 5.38E-03 |  |
| biological process | GO:0046128 | purine ribonucleoside metabolic process | 5.38E-03 |  |
| biological process | GO:0019438 | aromatic compound biosynthetic process | 5.50E-03 |  |
| biological process | GO:0051173 | positive regulation of nitrogen compound metabolic process | 5.53E-03 | # |
| biological process | GO:0018130 | heterocycle biosynthetic process | 5.74E-03 | # |
| biological process | GO:0006091 | generation of precursor metabolites and energy | 6.25E-03 | # |
| biological process | GO:0008104 | protein localization | 6.38E-03 |  |
| biological process | GO:0008380 | RNA splicing | 6.72E-03 |  |
| biological process | GO:0045184 | establishment of protein localization | 6.83E-03 | # |
| biological process | GO:0090304 | nucleic acid metabolic process | 7.06E-03 |  |
| biological process | GO:0006397 | mRNA processing | 7.20E-03 |  |
| biological process | GO:0009987 | cellular process | 8.05E-03 | # |
| biological process | GO:0007165 | signal transduction | 8.14E-03 | # |
| biological process | GO:0006006 | glucose metabolic process | 8.44E-03 | # |
| biological process | GO:0006508 | proteolysis | 8.77E-03 | # |
| biological process | GO:0000377 | RNA splicing, via transesterification reactions with bulged adenosine as nucleophile | 9.27E-03 |  |
| biological process | GO:0016093 | polyprenol metabolic process | 9.57E-03 |  |
| biological process | GO:0000375 | RNA splicing, via transesterification reactions | 1.00E-02 |  |
| biological process | GO:0015031 | protein transport | 1.03E-02 | # |
| biological process | GO:0034654 | nucleobase-containing compound biosynthetic process | 1.06E-02 | # |
| biological process | GO:0045333 | cellular respiration | 1.11E-02 |  |
| biological process | GO:0009119 | ribonucleoside metabolic process | 1.12E-02 |  |
| biological process | GO:1901575 | organic substance catabolic process | 1.12E-02 |  |
| biological process | GO:0019318 | hexose metabolic process | 1.37E-02 | # |
| biological process | GO:0009116 | nucleoside metabolic process | 1.42E-02 |  |
| biological process | GO:1901657 | glycosyl compound metabolic process | 1.63E-02 |  |
| biological process | GO:0010257 | NADH dehydrogenase complex assembly | 1.79E-02 |  |
| biological process | GO:0044723 | single-organism carbohydrate metabolic process | 1.93E-02 |  |
| biological process | GO:0005996 | monosaccharide metabolic process | 2.15E-02 | # |
| biological process | GO:0055114 | oxidation-reduction process | 2.20E-02 | # |
| biological process | GO:0006352 | DNA-dependent transcription, initiation | 2.35E-02 | # |
| biological process | GO:0006487 | protein N-linked glycosylation | 2.35E-02 |  |
| biological process | GO:0007049 | cell cycle | 2.35E-02 |  |
| biological process | GO:0071702 | organic substance transport | 2.93E-02 |  |
| biological process | GO:0022402 | cell cycle process | 3.10E-02 | # |
| biological process | GO:0006013 | mannose metabolic process | 3.13E-02 |  |
| biological process | GO:0046500 | S-adenosylmethionine metabolic process | 3.13E-02 |  |
| biological process | GO:0050680 | negative regulation of epithelial cell proliferation | 3.13E-02 |  |
| biological process | GO:0006289 | nucleotide-excision repair | 3.13E-02 | # |
| biological process | GO:0019058 | viral infectious cycle | 3.13E-02 | # |
| biological process | GO:0007186 | G-protein coupled receptor signaling pathway | 3.28E-02 | # |
| biological process | GO:1901564 | organonitrogen compound metabolic process | 3.45E-02 |  |
| biological process | GO:0009896 | positive regulation of catabolic process | 3.46E-02 | # |
| biological process | GO:0019080 | viral genome expression | 3.46E-02 |  |
| biological process | GO:0045935 | positive regulation of nucleobase-containing compound metabolic process | 3.46E-02 | # |
| biological process | GO:0065008 | regulation of biological quality | 3.52E-02 | # |
| biological process | GO:0016032 | viral reproduction | 3.89E-02 |  |
| biological process | GO:0022415 | viral reproductive process | 3.89E-02 | # |
| biological process | GO:0031016 | pancreas development | 3.89E-02 |  |
| biological process | GO:0009056 | catabolic process | 3.89E-02 | # |
| biological process | GO:0009057 | macromolecule catabolic process | 3.91E-02 | # |
| biological process | GO:0051186 | cofactor metabolic process | 4.10E-02 |  |
| biological process | GO:0044764 | multi-organism cellular process | 4.32E-02 |  |
| biological process | GO:0006351 | transcription, DNA-dependent | 4.33E-02 | # |
| biological process | GO:0032774 | RNA biosynthetic process | 4.33E-02 | # |
| biological process | GO:0022900 | electron transport chain | 4.40E-02 | # |
| biological process | GO:0051649 | establishment of localization in cell | 4.40E-02 | # |
| cellular component | GO:0044424 | intracellular part | 5.00E-14 | # |
| cellular component | GO:0005622 | intracellular | 5.00E-14 | # |
| cellular component | GO:0043226 | organelle | 5.00E-14 | # |
| cellular component | GO:0043229 | intracellular organelle | 5.00E-14 | # |
| cellular component | GO:0043227 | membrane-bounded organelle | 5.00E-14 | # |
| cellular component | GO:0043231 | intracellular membrane-bounded organelle | 5.00E-14 | # |
| cellular component | GO:0005623 | cell | 1.78E-13 | # |
| cellular component | GO:0044464 | cell part | 1.78E-13 | # |
| cellular component | GO:0032991 | macromolecular complex | 3.64E-10 | # |
| cellular component | GO:0044422 | organelle part | 1.59E-09 | # |
| cellular component | GO:0044446 | intracellular organelle part | 1.44E-07 | # |
| cellular component | GO:0030529 | ribonucleoprotein complex | 2.54E-07 | # |
| cellular component | GO:0044444 | cytoplasmic part | 1.12E-06 | # |
| cellular component | GO:0005634 | nucleus | 1.47E-06 | # |
| cellular component | GO:0005737 | cytoplasm | 1.91E-06 | # |
| cellular component | GO:0031967 | organelle envelope | 3.21E-06 | # |
| cellular component | GO:0005743 | mitochondrial inner membrane | 4.60E-06 | # |
| cellular component | GO:0044428 | nuclear part | 1.12E-05 | # |
| cellular component | GO:0019866 | organelle inner membrane | 2.09E-05 | # |
| cellular component | GO:0031975 | envelope | 4.45E-05 | # |
| cellular component | GO:0031090 | organelle membrane | 6.17E-05 | # |
| cellular component | GO:0031966 | mitochondrial membrane | 6.79E-05 | # |
| cellular component | GO:0044429 | mitochondrial part | 9.50E-05 | # |
| cellular component | GO:0005740 | mitochondrial envelope | 1.13E-04 | # |
| cellular component | GO:0031981 | nuclear lumen | 1.47E-04 | # |
| cellular component | GO:0043233 | organelle lumen | 1.47E-04 | # |
| cellular component | GO:0070013 | intracellular organelle lumen | 1.47E-04 | # |
| cellular component | GO:0031974 | membrane-enclosed lumen | 2.23E-04 | # |
| cellular component | GO:0005739 | mitochondrion | 2.66E-04 | # |
| cellular component | GO:0043234 | protein complex | 2.66E-04 | # |
| cellular component | GO:0000502 | proteasome complex | 5.30E-03 | # |
| cellular component | GO:0031224 | intrinsic to membrane | 5.30E-03 | # |
| cellular component | GO:0044451 | nucleoplasm part | 1.75E-02 | # |
| cellular component | GO:0071944 | cell periphery | 1.91E-02 |  |
| cellular component | GO:0070469 | respiratory chain | 2.20E-02 |  |
| cellular component | GO:0005886 | plasma membrane | 2.31E-02 | # |
| cellular component | GO:0044425 | membrane part | 2.63E-02 | # |
| cellular component | GO:0005654 | nucleoplasm | 2.78E-02 | # |
| cellular component | GO:0031201 | SNARE complex | 3.28E-02 | # |
| cellular component | GO:0044391 | ribosomal subunit | 3.83E-02 |  |
| cellular component | GO:0005746 | mitochondrial respiratory chain | 4.04E-02 | # |
| cellular component | GO:0005840 | ribosome | 4.33E-02 | # |
| cellular component | GO:0044455 | mitochondrial membrane part | 4.40E-02 | # |
| cellular component | GO:0005839 | proteasome core complex | 4.51E-02 | # |
| molecular function | GO:0038023 | signaling receptor activity | 6.92E-08 |  |
| molecular function | GO:0004888 | transmembrane signaling receptor activity | 9.62E-08 | # |
| molecular function | GO:0004872 | receptor activity | 9.62E-08 | # |
| molecular function | GO:0004871 | signal transducer activity | 1.84E-06 | # |
| molecular function | GO:0060089 | molecular transducer activity | 8.30E-06 | # |
| molecular function | GO:0005198 | structural molecule activity | 5.06E-04 | # |
| molecular function | GO:0005216 | ion channel activity | 3.72E-03 | # |
| molecular function | GO:0022838 | substrate-specific channel activity | 3.72E-03 | # |
| molecular function | GO:0015267 | channel activity | 3.72E-03 | # |
| molecular function | GO:0022803 | passive transmembrane transporter activity | 3.72E-03 | # |
| molecular function | GO:0015075 | ion transmembrane transporter activity | 1.03E-02 | # |
| molecular function | GO:0003676 | nucleic acid binding | 1.14E-02 | # |
| molecular function | GO:0022836 | gated channel activity | 1.37E-02 | # |
| molecular function | GO:0004661 | protein geranylgeranyltransferase activity | 1.42E-02 |  |
| molecular function | GO:0008320 | protein transmembrane transporter activity | 1.42E-02 |  |
| molecular function | GO:0022884 | macromolecule transmembrane transporter activity | 1.42E-02 |  |
| molecular function | GO:0004519 | endonuclease activity | 1.44E-02 |  |
| molecular function | GO:0015276 | ligand-gated ion channel activity | 1.52E-02 | # |
| molecular function | GO:0022834 | ligand-gated channel activity | 1.52E-02 | # |
| molecular function | GO:0022839 | ion gated channel activity | 1.75E-02 | # |
| molecular function | GO:0016765 | transferase activity, transferring alkyl or aryl (other than methyl) groups | 2.20E-02 | # |
| molecular function | GO:0022857 | transmembrane transporter activity | 2.29E-02 | # |
| molecular function | GO:0004518 | nuclease activity | 3.01E-02 |  |
| molecular function | GO:0022891 | substrate-specific transmembrane transporter activity | 3.13E-02 | # |
| molecular function | GO:0000030 | mannosyltransferase activity | 3.45E-02 |  |
| molecular function | GO:0008318 | protein prenyltransferase activity | 3.45E-02 |  |
| molecular function | GO:0008565 | protein transporter activity | 3.45E-02 |  |
| molecular function | GO:0005488 | binding | 3.52E-02 | # |
| molecular function | GO:0005215 | transporter activity | 3.67E-02 | # |
| molecular function | GO:0008757 | S-adenosylmethionine-dependent methyltransferase activity | 3.69E-02 | # |
| molecular function | GO:0016853 | isomerase activity | 4.33E-02 | # |
| molecular function | GO:0004672 | protein kinase activity | 4.48E-02 | # |
| molecular function | GO:0016861 | intramolecular oxidoreductase activity, interconverting aldoses and ketoses | 4.51E-02 |  |

**Table S6. List of the enriched GO terms for particularly fast-evolving genes (identified by the top 5% *Ka*) in *Latimeria chalumnae* vs. *Danio rerio*.**

| Gene ontology | GO term id | GO term description | FDR (BH) | Flag (immune-related GO terms) |
| --- | --- | --- | --- | --- |
| biological process | GO:0006952 | defense response | 0.000112315 | # |
| biological process | GO:0002376 | immune system process | 0.00146689 | # |
| biological process | GO:0006950 | response to stress | 0.00270781 | # |
| biological process | GO:0051179 | localization | 0.006140767 | # |
| biological process | GO:0008152 | metabolic process | 0.018355275 | # |
| biological process | GO:0044237 | cellular metabolic process | 0.019024157 | # |
| biological process | GO:0051234 | establishment of localization | 0.019024157 | # |
| biological process | GO:0012501 | programmed cell death | 0.0333976 | # |
| biological process | GO:0008219 | cell death | 0.038087222 | # |
| biological process | GO:0016265 | death | 0.038087222 | # |
| biological process | GO:0006810 | transport | 0.039973105 | # |
| cellular component | GO:0005576 | extracellular region | 0.012750418 | # |
| cellular component | GO:0044421 | extracellular region part | 0.038087222 | # |
| molecular function | GO:0005102 | receptor binding | 0.000112315 | # |
| molecular function | GO:0003824 | catalytic activity | 0.00270781 | # |
| molecular function | GO:0004872 | receptor activity | 0.004706432 | # |
| molecular function | GO:0005125 | cytokine activity | 0.004721433 | # |
| molecular function | GO:0005126 | cytokine receptor binding | 0.004721433 | # |
| molecular function | GO:0030246 | carbohydrate binding | 0.012750418 | # |
| molecular function | GO:0004888 | transmembrane signaling receptor activity | 0.0425896 | # |
| molecular function | GO:0038023 | signaling receptor activity | 0.046835429 |  |

**Table S7. List of the enriched GO terms for moderately fast-evolving genes (identified by the top 10% *Ka*) in *Latimeria chalumnae* vs. *Danio rerio*.**

| Gene ontology | GO term id | GO term description | FDR (BH) | Flag (immune-related GO terms) |
| --- | --- | --- | --- | --- |
| biological process | GO:0001910 | regulation of leukocyte mediated cytotoxicity | 0.024681244 | # |
| biological process | GO:0001912 | positive regulation of leukocyte mediated cytotoxicity | 0.024681244 | # |
| biological process | GO:0031341 | regulation of cell killing | 0.024681244 | # |
| biological process | GO:0031343 | positive regulation of cell killing | 0.024681244 | # |
| biological process | GO:0002705 | positive regulation of leukocyte mediated immunity | 0.031185455 | # |
| biological process | GO:0002699 | positive regulation of immune effector process | 0.032914286 | # |
| biological process | GO:0002703 | regulation of leukocyte mediated immunity | 0.032914286 | # |
| biological process | GO:0051179 | localization | 0.039774316 | # |
| biological process | GO:0006952 | defense response | 0.0414208 | # |
| biological process | GO:0007599 | hemostasis | 0.042030546 |  |
| biological process | GO:0050878 | regulation of body fluid levels | 0.049541565 | # |
| cellular component | GO:0005576 | extracellular region | 0.024681244 | # |
| cellular component | GO:0044424 | intracellular part | 0.024681244 | # |
| cellular component | GO:0005622 | intracellular | 0.031185455 | # |
| cellular component | GO:0044421 | extracellular region part | 0.032914286 | # |
| cellular component | GO:0043227 | membrane-bounded organelle | 0.038656 | # |
| molecular function | GO:0005102 | receptor binding | 3.53792E-05 | # |
| molecular function | GO:0005126 | cytokine receptor binding | 0.00021312 | # |
| molecular function | GO:0005125 | cytokine activity | 0.004237653 | # |
| molecular function | GO:0003824 | catalytic activity | 0.036078933 | # |
| molecular function | GO:0005496 | steroid binding | 0.039774316 | # |
| molecular function | GO:0032813 | tumor necrosis factor receptor superfamily binding | 0.039774316 | # |
| molecular function | GO:0005515 | protein binding | 0.042030546 | # |

**Table S8. List of the enriched GO terms for particularly fast-evolving genes (identified by the top 5% *Ka*) in *Takifugu rubripes* vs. *Danio rerio*.**

| Gene ontology | GO term id | GO term description | FDR (BH) | Flag  (immune-related GO terms) | |
| --- | --- | --- | --- | --- | --- |
| biological process | GO:0002252 | immune effector process | 0.037161115 | | # |
| biological process | GO:0002253 | activation of immune response | 0.037161115 | | # |
| biological process | GO:0002376 | immune system process | 7.80498E-05 | | # |
| biological process | GO:0006810 | transport | 0.02379228 | | # |
| biological process | GO:0006952 | defense response | 0.037161115 | | # |
| biological process | GO:0007051 | spindle organization | 0.046115774 | |  |
| biological process | GO:0007599 | hemostasis | 0.00041946 | |  |
| biological process | GO:0008152 | metabolic process | 0.024238227 | | # |
| biological process | GO:0009607 | response to biotic stimulus | 0.026335188 | | # |
| biological process | GO:0043207 | response to external biotic stimulus | 0.024538133 | |  |
| biological process | GO:0050776 | regulation of immune response | 0.046115774 | | # |
| biological process | GO:0050778 | positive regulation of immune response | 0.043315345 | | # |
| biological process | GO:0050878 | regulation of body fluid levels | 0.000434603 | | # |
| biological process | GO:0051179 | localization | 0.003242381 | | # |
| biological process | GO:0051234 | establishment of localization | 0.0044023 | | # |
| biological process | GO:0051707 | response to other organism | 0.024538133 | | # |
| biological process | GO:0065008 | regulation of biological quality | 0.040098286 | | # |
| cellular component | GO:0005576 | extracellular region | 0.026363177 | | # |
| cellular component | GO:0042611 | MHC protein complex | 0.024538133 | | # |
| molecular function | GO:0000166 | nucleotide binding | 0.037161115 | |  |
| molecular function | GO:0003824 | catalytic activity | 0.000429175 | | # |
| molecular function | GO:0004857 | enzyme inhibitor activity | 0.046122781 | | # |
| molecular function | GO:0004872 | receptor activity | 0.024538133 | | # |
| molecular function | GO:0004888 | transmembrane signaling receptor activity | 0.037161115 | | # |
| molecular function | GO:0004896 | cytokine receptor activity | 0.004670778 | | # |
| molecular function | GO:0005102 | receptor binding | 7.80498E-05 | | # |
| molecular function | GO:0005126 | cytokine receptor binding | 7.80498E-05 | | # |
| molecular function | GO:0005179 | hormone activity | 0.037161115 | | # |
| molecular function | GO:0005515 | protein binding | 0.037635926 | | # |
| molecular function | GO:0036094 | small molecule binding | 0.036943278 | |  |
| molecular function | GO:0038023 | signaling receptor activity | 0.037161115 | |  |
| molecular function | GO:1901265 | nucleoside phosphate binding | 0.037161115 | |  |

**Table S9. List of the enriched GO terms for moderately fast-evolving genes (identified by the top 10% *Ka*) in *Takifugu rubripes* vs. *Danio rerio*.**

| Gene ontology | GO term id | GO term description | FDR (BH) | Flag (immune-related GO terms) |
| --- | --- | --- | --- | --- |
| biological process | GO:0044281 | small molecule metabolic process | 3.56E-05 |  |
| biological process | GO:0044710 | single-organism metabolic process | 6.84E-04 |  |
| biological process | GO:0006810 | transport | 6.84E-04 | # |
| biological process | GO:0051234 | establishment of localization | 7.36E-04 | # |
| biological process | GO:0002376 | immune system process | 1.14E-03 | # |
| biological process | GO:1902578 | single-organism localization | 1.15E-03 |  |
| biological process | GO:0044765 | single-organism transport | 1.52E-03 |  |
| biological process | GO:0051179 | localization | 1.54E-03 | # |
| biological process | GO:0044267 | cellular protein metabolic process | 1.65E-03 | # |
| biological process | GO:0044237 | cellular metabolic process | 3.20E-03 | # |
| biological process | GO:0008152 | metabolic process | 3.21E-03 | # |
| biological process | GO:0007599 | hemostasis | 4.80E-03 |  |
| biological process | GO:0050878 | regulation of body fluid levels | 6.11E-03 | # |
| biological process | GO:0044238 | primary metabolic process | 7.58E-03 | # |
| biological process | GO:0006811 | ion transport | 9.73E-03 |  |
| biological process | GO:0009056 | catabolic process | 2.82E-02 | # |
| biological process | GO:0010952 | positive regulation of peptidase activity | 2.98E-02 |  |
| biological process | GO:0045862 | positive regulation of proteolysis | 2.98E-02 |  |
| biological process | GO:0019538 | protein metabolic process | 3.49E-02 | # |
| biological process | GO:0071704 | organic substance metabolic process | 3.49E-02 |  |
| biological process | GO:0006952 | defense response | 4.12E-02 | # |
| biological process | GO:0051726 | regulation of cell cycle | 4.28E-02 | # |
| biological process | GO:0006796 | phosphate-containing compound metabolic process | 4.28E-02 | # |
| cellular component | GO:0005576 | extracellular region | 1.34E-04 | # |
| cellular component | GO:0032991 | macromolecular complex | 3.20E-03 | # |
| cellular component | GO:0044421 | extracellular region part | 4.80E-03 | # |
| cellular component | GO:0042611 | MHC protein complex | 1.08E-02 | # |
| cellular component | GO:0044444 | cytoplasmic part | 2.36E-02 | # |
| molecular function | GO:0003824 | catalytic activity | 1.26E-05 | # |
| molecular function | GO:0004872 | receptor activity | 3.88E-05 | # |
| molecular function | GO:0005102 | receptor binding | 4.22E-04 | # |
| molecular function | GO:0005126 | cytokine receptor binding | 6.84E-04 | # |
| molecular function | GO:0004888 | transmembrane signaling receptor activity | 7.94E-04 | # |
| molecular function | GO:0038023 | signaling receptor activity | 9.74E-04 |  |
| molecular function | GO:0005215 | transporter activity | 1.59E-03 | # |
| molecular function | GO:0004896 | cytokine receptor activity | 2.03E-03 | # |
| molecular function | GO:0022892 | substrate-specific transporter activity | 2.14E-03 | # |
| molecular function | GO:0022857 | transmembrane transporter activity | 2.99E-03 | # |
| molecular function | GO:0097367 | carbohydrate derivative binding | 4.80E-03 |  |
| molecular function | GO:0016491 | oxidoreductase activity | 4.80E-03 | # |
| molecular function | GO:0004871 | signal transducer activity | 5.14E-03 | # |
| molecular function | GO:0036094 | small molecule binding | 5.64E-03 |  |
| molecular function | GO:0017076 | purine nucleotide binding | 5.74E-03 |  |
| molecular function | GO:0000166 | nucleotide binding | 5.76E-03 |  |
| molecular function | GO:1901265 | nucleoside phosphate binding | 5.76E-03 |  |
| molecular function | GO:0032553 | ribonucleotide binding | 6.62E-03 |  |
| molecular function | GO:0032555 | purine ribonucleotide binding | 6.62E-03 |  |
| molecular function | GO:0005179 | hormone activity | 9.73E-03 | # |
| molecular function | GO:0016773 | phosphotransferase activity, alcohol group as acceptor | 1.15E-02 | # |
| molecular function | GO:0004672 | protein kinase activity | 2.19E-02 | # |
| molecular function | GO:0016817 | hydrolase activity, acting on acid anhydrides | 2.19E-02 | # |
| molecular function | GO:0017111 | nucleoside-triphosphatase activity | 2.58E-02 | # |
| molecular function | GO:0016538 | cyclin-dependent protein serine/threonine kinase regulator activity | 2.65E-02 |  |
| molecular function | GO:0016462 | pyrophosphatase activity | 2.82E-02 | # |
| molecular function | GO:0016818 | hydrolase activity, acting on acid anhydrides, in phosphorus-containing anhydrides | 2.84E-02 | # |
| molecular function | GO:0032559 | adenyl ribonucleotide binding | 3.49E-02 |  |
| molecular function | GO:0030554 | adenyl nucleotide binding | 3.49E-02 |  |
| molecular function | GO:0060089 | molecular transducer activity | 4.28E-02 | # |
| molecular function | GO:0061135 | endopeptidase regulator activity | 4.28E-02 |  |
| molecular function | GO:0005515 | protein binding | 4.41E-02 | # |
| molecular function | GO:0016740 | transferase activity | 4.53E-02 | # |

**Table S10.** **Downloaded information of gene sets used in this study.**

| Scientific names | English names | Versions | Download link |
| --- | --- | --- | --- |
| *Branchistoma floridae* | Florida amphixous | Version 2 | http://genome.jgi-psf.org/Brafl1/Brafl1.home.html |
| *Branchistoma belcheri* | Chinese amphixous | V18h27.r3 | http://genome.bucm.edu.cn/lancelet/download_data.php |
| *Danio rerio* | Zebrafish | Ensembl v86.0 | http://www.ensembl.org/info/data/ftp/index.html |
| *Latimeria chalumnae* | Coelacanth | Ensembl v86.0 | http://www.ensembl.org/info/data/ftp/index.html |
| *Takifugu rubripes* | Japanese pufferfish | Ensembl v86.0 | http://www.ensembl.org/info/data/ftp/index.html |

**Table S11. Primers used in quantitative RT-PCRs.**

| Gene name | Gene ID | Sequence (5'-3') | Size (bp) |
| --- | --- | --- | --- |
| *S20* | 107260 F | F: ACTCAGATCCACCGCATCA | 112 |
|  |  | R: CCTTCACCTGGAGGTTCTTCT |  |
| *HMGCL* | 143780R | F: CTATGTTGGAGGCTGTGA | 102 |
|  |  | R: GTAGTGCGGTGAGTATGT |  |
| *SAT2* | 047790R | F: TCCAGAGCAAGTCAAGAT | 147 |
|  |  | R: AAGAAGTACATGGCATAGC |  |
| *CYBS* | 145260R | F: TTGAGCAGATTGTGACAGA | 117 |
|  |  | R: CGTTGTAGTTAAAGTAGCAGAG |  |
| *MDH2* | 004230R | F: GAGACACTGGACAATCTGACC | 167 |
|  |  | R: CCACATTCAATCACTCCTTCCT |  |
